# CSNK2 in cancer: pathophysiology and translational applications

**DOI:** 10.1038/s41416-021-01616-2

**Published:** 2021-11-12

**Authors:** Scott W. Strum, Laszlo Gyenis, David W. Litchfield

**Affiliations:** 1 Department of Medicine, Western University, Schulich School of Medicine & Dentistry, London, ON Canada; 2 Department of Biochemistry, Western University, Schulich School of Medicine & Dentistry, London, ON Canada; 3 Department of Biochemistry & Department of Oncology, Western University, Schulich School of Medicine & Dentistry, London, ON Canada

**Keywords:** Targeted therapies, Kinases

## Abstract

Protein kinase CSNK2 (CK2) is a pleiotropic serine/threonine kinase frequently dysregulated in solid and hematologic malignancies. To consolidate a wide range of biological and clinically oriented data from this unique kinase in cancer, this systematic review summarises existing knowledge from in vitro, in vivo and pre-clinical studies on CSNK2 across 24 different human cancer types. CSNK2 mRNA transcripts, protein levels and activity were found to be routinely upregulated in cancer, and commonly identified phosphotargets included AKT, STAT3, RELA, PTEN and TP53. Phenotypically, it frequently influenced evasion of apoptosis, enhancement of proliferation, cell invasion/metastasis and cell cycle control. Clinically, it held prognostic significance across 14 different cancers, and its inhibition in xenograft experiments resulted in a positive treatment response in 12. In conjunction with commentary on preliminary studies of CSNK2 inhibitors in humans, this review harmonises an extensive body of CSNK2 data in cancer and reinforces its emergence as an attractive target for cancer therapy. Continuing to investigate CSNK2 will be crucial to advancing our understanding of CSNK2 biology, and offers the promise of important new discoveries scientifically and clinically.

## Introduction

Phosphorylation is the most common type of reversible post-translational modification in living cells [1], with protein kinases representing the enzymes that catalyse these phosphorylation events. Encoded by over 500 genes in the human genome [2, 3], protein kinases are estimated to phosphorylate well over 30% of all cellular proteins [4, 5]. They can be classified based on the sequence homology of their catalytic domains into the following major groups: (1) Serine/Threonine kinases: *AGC, CAMK, CMGC, STE, CK1*; (2) Tyrosine kinases: *TK, RGC* and (3) TK-like kinases [6]. Protein kinases influence a wide variety of cellular functions including apoptosis, motility, growth, differentiation, proliferation and angiogenesis. Dysregulation of any one of these pathways can lead to significant maladaptation with outcomes ranging from cell death to neoplastic transformation.

As a class, protein kinases play important roles in health and disease [7]. In particular, they have been observed to influence the development of cancer, impacting virtually all aspects of oncogenesis [8]. This has led to the development of targeted therapies that have yielded significant advances in cancer treatment. To date, over 30 targeted therapies have been approved by the FDA to treat cancer covering all three kinase groups [9]. And ever since the first oncogene was discovered to be a kinase [10], increasing efforts have been made to develop small-molecule inhibitors targeting these proteins. A classic example of this is evidenced by the drug imatinib (Gleevec), proven to be very effective in the treatment of CML. Borne from targeting the BCR-Abl gene mutation highly prevalent in this malignancy, imatinib functions as a tyrosine kinase inhibitor that dramatically increases 5-year survival rates to around 89% [11], compared to ~30% without treatment [12]. Although efficacious in this setting, the kinase inhibition imparted by imatinib is not specific to BCR-Abl, which has subsequently been capitalised on to treat a series of other conditions such as systemic mastocytosis and KIT-positive GIST tumours [13].

Despite advances such as these, however, cancer often still holds a devastating prognosis. In 2018 it remained the second leading cause of death globally [14]. It is imperative that new treatment options become available to help broaden the armamentarium of therapies available to manage diseases that continue to have limited treatment options. One such targetable class of protein kinases that has been of growing interest over the last several decades has been the serine/threonine kinase CK2 (CSNK2).

Functionally crucial for cell development and commonly dysregulated in cancer, CSNK2 is a pleiotropic serine/threonine kinase encoded by two distinct catalytic isoforms (CSNK2A1 and CSNK2A2) that can form complexes with a regulatory subunit (CSNK2B). Belonging to the CMGC group of kinases, CSNK2 functions primarily as part of a tetrameric complex with two regulatory CSNK2B subunits, but it can also function independently in monomeric form. Each of the CSNK2A subunits is constitutively catalytically active, but their association with CSNK2B can change its functional properties such as its substrate specificity [15, 16], as well as subcellular localisation [17, 18]. Further, independent functions of the CSNK2B subunit have also been described [16, 19, 20].

CSNK2 kinase subunits have a unique minimum consensus sequence for phosphorylation of Ser-X-X-acidic [21], largely distinct from many other protein kinases [16]. They are generally found in the cytoplasm and nucleus, whose distribution varies between cell lines as well as under different physiologic conditions [22–24]. Its functionality is broad, potentially responsible for about 10% of the phosphoproteome based on the prevalence of phosphopeptides that conform to the CSNK2 recognition motif [25]. It is no surprise that its activity spans a multitude of signaling pathways including Wnt [26], JAK-STAT [27], PI3K/AKT [28, 29] and numerous others. As a result, its regulation becomes paramount in defining its roles in different biological contexts.

Regulatory mechanisms of CSNK2 are varied and remain relatively poorly understood, due at least in part to its constitutive activity when assayed in vitro. What is known of its regulation has been reviewed in Olsten et al 2005 [30] and Litchfield et al. 2003 [16]. Specific to CSNK2 in cancer, regulatory mechanisms previously studied have included localisation [31], scaffolding [32], regulation of CSNK2 subunit expression/assembly/localisation [33], post-translational modification [34] and small-molecule interactions [35]. Functionally, CSNK2 plays an important role in modulating cellular processes such as proliferation [21], signaling pathway activation [36], apoptosis [37], angiogenesis [38], growth [16] and metabolism [39]. Crucial for embryonic development in mouse models [40–42], dysregulation of CSNK2 can also lead to a series of disease states including inflammation [43], cardiomyopathy [44] and cancer.

Given the high incidence of cancer mortality worldwide [14], CSNK2 is an ideal target for further research as in vitro and in vivo studies have repeatedly shown it is dysregulated in both hematologic and solid malignancies [45, 46]. Notably, Seldin and colleagues have shown that overexpression of CSNK2A1 leads to a stochastic propensity to developing lymphoma in mice [47]. Several other studies have reported its tumorigenic potential through upregulation of CSNK2 subunits as well [48–50]. Discoveries such as these, in combination with its widespread dysregulation in cancer, have made it a protein of interest not only for biological study, but also for disease management.

With growing evidence that CSNK2 plays an important role in cancer, efforts have been made over the last twenty years to target it for drug development. This has been facilitated in the part through the generation of CSNK2-specific inhibitors CX4945 and CIGB-300. CX4945 was developed in 2011 as the result of a structure-based optimzation of a candidate inhibitor that was highly selective, orally bioavailable, and demonstrated promise in preliminary xenograft experiments [51]. Its use has since expanded widely, and it was granted orphan drug status by the FDA in 2017 for the treatment of cholangiocarcinoma. Another notable CSNK2 inhibitor is CIGB-300, identified as the result of screening a random cyclic peptide phage display library for candidate drugs [52]. It has similarly since been used in numerous pre-clinical settings. In keeping with the paradigm shift of cancer treatment whereby isolated drivers of oncogenesis are specifically targeted for treatment (eg CDK4/6 inhibitors, MEK inhibitors, VEGF inhibitors), it is foreseeable that inhibitors of CSNK2 such as these may well represent the next wave of clinically efficacious small-molecule therapeutics.

Building on the framework outlined above, this systematic review provides a summary of existing knowledge from in vitro and in vivo studies on CSNK2 mRNA/protein expression and activity levels, downstream phosphotargets, associated phenotypes, in vivo, and pre-clinical studies across a wide range of 24 different cancer types. Major trends are highlighted, balanced with commentary on the biologic complexity of this kinase. These results are then integrated to identify high-yield areas of CSNK2 inhibition that hold promise for therapeutic benefit. Our analysis supports CSNK2 as a potentially attractive target for cancer therapy and points to specific areas in which further investigation will be critical to advance our understanding of the biology and pathophysiology of CSNK2.

## Pathophysiology of CSNK2 in cancer

CSNK2 is a constitutively active pleiotropic protein kinase involved in many homeostatic cellular processes, such as proliferation and cell division. Crucial for cell development, it has also been found to be frequently dysregulated in cancer [45, 46]. Interestingly, despite this dysregulation, CSNK2 tends not to be heavily mutated in malignant cells. Using CBioPortal, CSNK2A1, CSNK2A2 and CSNK2B were found to harbor somatic mutation frequencies of only 0.8%, 0.6% and <0.1%, respectively, the majority of which were found to occur with very low counts [53, 54]. Using the COSMIC [55] and CBioPortal databases [53, 54] along with CSNK2A1 as an example kinase subunit, mutations were identified across nearly the entire protein sequence, including sites within the nucleotide binding cluster, acidic loop, as well as the CSNK2B binding region. By contrast, the most frequent mutations were not found within any of these functional areas. Thus, the effect these mutations have on CSNK2 in oncogenesis remains uncertain, but may suggest they play a more minor role in its oncogenic potential than other factors.

More aptly, CSNK2 has demonstrated itself to be a central player in malignant cell biology through ‘non-oncogene addiction’ [56]. In non-oncogene addiction, dysregulation of a target protein helps sustain the activities necessary for cancer cell propagation. The pathophysiologic mechanisms that allow CSNK2 to drive these hallmarks of oncogenesis are therefore imperative to examine. We herein describe the overarching findings from a systematic review of the literature of CSNK2 targets identified in 24 unique cancers, extracting data on mRNA transcript, protein, kinase activity levels, phosphotargets, phenotypic behaviours and in vivo experiments. Detailed in the Supplementary Materials, data compiled in this review were collected from records of all years in PubMed up until May 2020 using PRISMA methodology (Appendix 1; Table 1 and Table 2 with corresponding references in Table S1 and Appendix 2; Table S2).

**Table 1:**
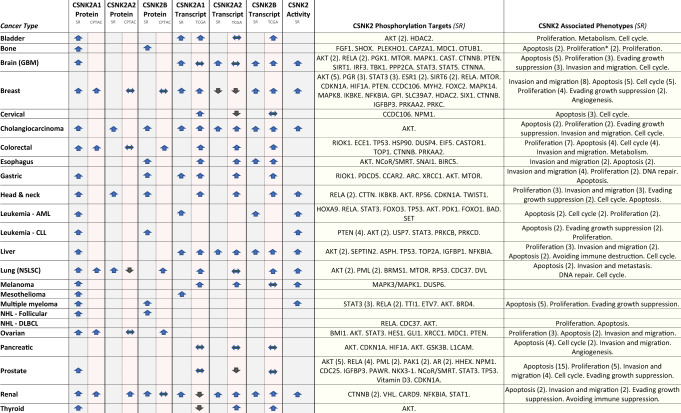
CSNK2 pathophysiologic data sorted by cancer type.

CSNK2 subunit transcript, protein and activity levels in cancer were reported as increased (up arrow), decreased (down arrow) or equivalent (sideways arrow). CSNK2 transcript and protein columns contained paired data: arrows in the left half of each column were data from publications identified in this systematic review (SR) (references in Table S1, with corresponding full citations in Appendix 2); arrows in the right half of each column represented external data from the TCGA or CPTAC, respectively (compiled by UALCAN [57]). All remaining data in Table 1 were derived strictly from this systematic review (SR). CSNK2 phosphotargets differentially regulated in cancer were listed, as well as CSNK2-associated cancer phenotypes. The number next to each phosphotarget or phenotype represented the number of supporting citations identified in this review (provided in brackets). Phosphotargets/phenotypes without a number were cited once.

^*^Proliferation correlated with decreased CSNK2 levels.

**Table 2:** CSNK2 inhibition in xenograft models sorted by cancer type.

Cancer Type	Engrafted Cell Line : Host	Intervention	Results (Control vs Intervention)	Reference (Appendix 2)
Bladder	EJ bladder cancer cells: nude mice (female)	EJ cells with either CSNK2A1 shRNA or control shRNA engrafted into nude mice	Tumour volume at 21 days: 425 mm^3^ vs 175 mm^3^ (*p* < 0.01)	237
Bone	143B cells: nude mice	CX4945 150 mg/kg daily oral	Survival lower (*p* < 0.05) and tumour size greater (*p* < 0.05) in control compared to CX4945 at study endpoints	193
Brain	U87-Luc cells: nude mice (female)	TBB 10 mg/kg daily intra-peritoneal	Intracranial luc fluorescence signal on day 11: 10^9.5^ vs 10^8.5^ (*p* < 0.01)	29
Brain	U3054MG cells: SCID or nude mice (female)	CX4945 75 mg/kg daily oral	Growth at 68 days: no significant difference between CX4945 and control	147
Brain	U87MG cells: nude mice	TBB 10 mg/kg q48 h intra-peritoneal	Tumour mass at 25 days: 650 mg vs 125 mg (*p* < 0.05)	42
Brain	LN229 cells: nude mice U87MG cells: nude mice	Anti-sense CSNK2A1 + cetuximab nanobioconjugate intra-venous twice per week for 3 weeks	LN229—survival: 37 days vs 70 days (*p* < 0.001) U87MG—survival: 34 days vs 48 days (*p* < 0.05)	31
Brain	U87MG cells: SCID mice	Engraftment of U87MG cells with CSNK2A knockdown via induced CSNK2A shRNA	Survival: 20 days vs >40 days (*p* < 0.0009)	137
Brain	X1046 cells: nude mice	CX4945 75 mg/kg BID oral for 28 days starting at day 5	Survival: 38 days vs 59 days	239
Breast	MDA-MB-231 cells: mice	Tenfibgen siRNA-CSNK2 0.01 mg/kg by tail vein injection	Tumour volume on day 10 relative to day 0: 2.1× vs 1.4× (*p* = 0.026)	200
Breast	MDA-MB-231 cells: nude mice (female)	Tenfibgen siRNA-CSNK2 0.01 mg/kg on day 1, 4, 7 by tail vein injection	Tumour volume on day 10 relative to day 0: 2.05× vs 1.35× (*p* < 0.05)	97
Breast	BT-474 cells: nude mice (female)	CX4945 75 mg/kg BID oral	Tumour volume at 30 days: 650 mm^3^ vs 190 mm^3^ (*p* < 0.001)	183
Cervical	SiHa cells: nude mice	CIGB-300 200ug daily intra-tumour for 5 days	Tumour volume at 21 days: 175 mm^3^ vs 60 mm^3^	245
Cervical	SiHa cells: nude mice (male and female)	CIGB-300 200ug daily intra-tumour for 5 days	Survival (median)—female: 33 days vs 59.0 days Survival (median)—male: 40.0 days vs 44.5 days	150
Head and neck	UM-SCC1 cells: SCID mice (female)	CX4945 75 mg/kg BID oral	Tumour volume at 25 days: 1000 mm^3^ vs 650 mm^3^ (*p* < 0.05)	13
Head and neck	FaDu cells: nude mice	Tenfibgen s50 RNAi-CSNK2 10 mg/kg twice q48h by tail vein injection	Tumour volume at 35 days: 1075 mm^3^ vs 75 mm^3^ (*p* < 0.0001)	204
Head and neck	(A) UM-SCC 11 A cells: SCID mice (male) (B) FaDu cells: nude mice (female)	(A) Tenfibgen nanocapsules with anti-CSNK2A1/A2 at 10 ug/kg intra-peritoneal q3days. (B) Tenfibgen nanocapsules with anti-CSNK2A1/A2 at 10 ug/kg IV q48 h for 2 doses	(A) Tumour volume at 7 days: 620 mm^3^ vs 250 mm^3^ (*p* < 0.01) (B) Tumour volume at 7 days: 180 mm^3^ vs 50 mm^3^ (*p* < 0.01)	18
Leukemia—CLL	MO1043 cells: nude mice	CX4945 75 mg/kg BID oral	Tumour volume at 13 days: 450 mm^3^ vs 225 mm^3^ (*p* < 0.001)	129
Leukemia—CLL	MO1043 cells: nude mice	CIGB-300 20 mg/kg intra-peritoneal for 5 days plus 2 days rest, then repeated	Tumour volume at 15 days: 1200 mm^3^ vs 600 mm^3^ (*p* < 0.001)	128
Liver	HepG2 cells: NMRI nude mice (male)	DMAT 500 ug/kg daily intra-peritoneal	Tumour volume at 10 days: 600 mm^3^ vs 200 mm^3^ (*P* < 0.05)	169
Lung	H-125 cells: nude mice (female)	P15-Tat (ie CIGB-300) 10 mg/kg intra-peritoneal for 5 days	Survival: 24 days vs 41 days (*p* = 0.0002)	151
Ovarian	SKOV3 EOC cells: nude mice	CX4945 75 mg/kg daily oral	Tumour volume at 21 days: 400 mm^3^ vs 180 mm^3^ (*p* < 0.01)	25
Ovarian	IGORV-1 cells: nude mice (female)	CX4945 75 mg/kg daily oral	Proliferative index at 42 days: 37% vs 16% (*p* < 0.001). Vascular tumur area at 42 days: 28% vs 14% (*p* < 0.001).	99
Ovarian	A2780 cells: nude mice (female)	CX4945 100 mg/kg BID oral (days 2, 5, 8 and 11) and/or gemcitabine 30 mg/kg intra-peritoneal every 3 days (days 1, 4, 7 and 10)	Time to reach tumour volume of 2000 mm^3^: 11 days (control), 13 days (CX4945), 37 days (gemcitabine), 51 days (combination)	182
Pancreatic	BxPC-3 cells: nude mice (female)	CX4945 75 mg/kg BID oral	Tumour volume at 35 days: 850 mm^3^ vs 190 mm^3^ (*p* < 0.001)	183
Pancreatic	MiaPaCa2 cells: nude mice (male)	siRNA PAK7 ± siRNA-CSNK2 q3days intra-peritoneal	Tumour volume at 21 days: 900 mm^3^ (control), 500 mm^3^ (PAK7) (*p* < 0.05), 230 mm^3^ (PAK7 + CSNK2) (*p* < 0.05)	57
Prostate	22Rv1 cells: SCID mice (male)	Tenfibgen RNAi-CSNK2 0.02 mg/kg by tail vein injection on days 1, 4, 7	Tumour weight on day 8: 1.1 g vs 0.35 g	201
Prostate	PC3-LN4 cells: mice	Tenfibgen RNAi-CSNK2 0.01 mg/kg by tail vein injection	Tumour volume on day 10 relative to day 0: 12.2× vs 5.2× (*p* = 0.005)	200
Prostate	PC3-LN4 cells: nude mice (male)	Tenfibgen RNAi-CSNK2 0.01 mg/kg by tail vein injection on days 1, 4, 7	Tumour volume fold change relative to day 0, at day 10: 12× vs 5× (*p* = 0.05)	3
Prostate	PC3-LN4: nude mice (male)	Tenfibgen RNAi-CSNK2 33 ng/kg intra-peritoneal twice, given 24 h apart	Tumour volume relative to control at 13 days: 100% vs 25% (*p* = 0.011)	203
Prostate	PC3-LN4: nude mice (male)	DMAT 500ug/kg daily intra-peritoneal for 6 days	Ki-67 proliferation index on day 7: 60% vs 30% (*p* < 0.002)	202
Prostate	PC3 cells: nude mice	TBB—dosing regimen not disclosed	Tumour volume at 35 days: 325 mm^3^ vs 125 mm^3^ (*p* < 0.001)	232
Prostate	PC3 cells: nude mice	CX4945 75 mg/kg BID oral	Tumour volume at 25 days: 775 mm^3^ vs 200 mm^3^	153
Prostate	PC3-LN4: nude mice (male)	Anti-sense CSNK2 16.5 ug/kg daily for 4 doses intra-peritoneal	Tumour mass at 13 days: 1000 mg vs 300 mg (*p* < 0.05)	199
Prostate	PC3-LN4: nude mice (male)	Anti-sense CSNK2A1 20 ug once intra-tumour	Tumour size at 8 days: 4.25 mm vs 0.0 mm	187

Representative experiments from each publication identified in this systematic review were highlighted. For each, the host organism (and sex, if specified) alongside the cancer cell line engrafted into the host was listed. Corresponding interventions were summarised, with results from the control and intervention groups recorded thereafter (absolute values were either quoted directly or their closest approximation listed). Statistical significance was provided when available. Numbered citations for all studies listed corresponded with references in Appendix 2 of the Supplementary Information.

Among the first data collected in this review were biochemical changes influenced by CSNK2 in cancer (Table 1). Elevations in CSNK2 activity, protein levels and mRNA levels in malignant tissues relative to their non-malignant counterparts were very common. To provide a comparator arm using large-scale high-throughput studies, parallel external mRNA transcript and protein data from The Cancer Genome Atlas (TCGA) and CPTAC (Clinical Proteomic Tumor Analysis Consortium), respectively, were compiled through UALCAN [57] and incorporated into Table 1. The trend of general upregulation remained evident across this vast cross-section of data. Thus, widespread CSNK2 dysregulation, combined with data from prior studies that overexpression of CSNK2A1 can lead to in vivo oncogenic transformation [48–50], strongly suggests an important role in cancer.

Variation in the data certainly did exist, however. For example, CSNK2A1 transcript levels were decreased in thyroid cancer cells yet corresponding CSNK2A1 protein levels were increased. In prostate cancer cells, CSNK2A1 transcript levels were unchanged, yet its protein levels were increased. Such examples reinforce the complexity of CSNK2 biology. Intracellular localisation [33, 58], post-translational modification [34] and small-molecule interactions [35] represent but some of the putative mechanisms that may account for these differences. Despite these variances, however, the overarching pattern was an elevation in CSNK2 activity, protein and transcript levels across a wide range of cancer types.

**Fig. 1:**
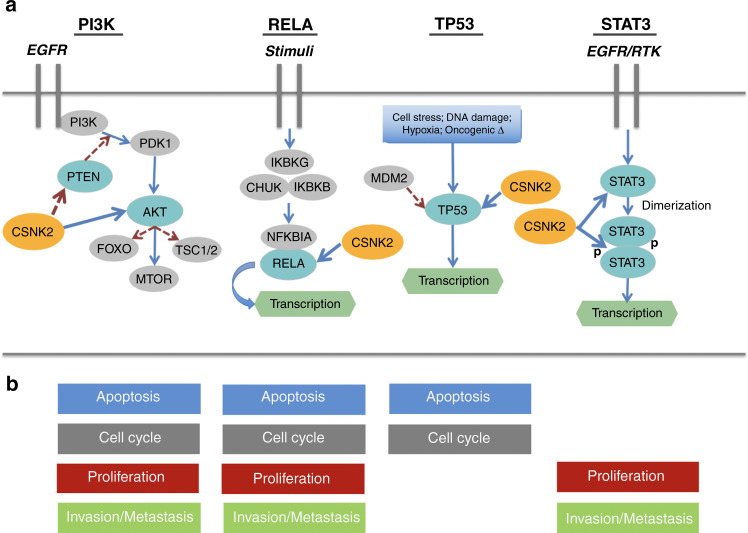
CSNK2 phosphotargets, associated pathways, and CSNK2-regulated phenotypes identified in this review. **a** Graphical summary of the pathways from which the five most commonly cited CSNK2 targets across all cancers were identified in this review: AKT, PTEN, RELA (NFkB), TP53 and STAT3. Red dotted arrows indicate negative regulation. Blue arrows indicate all other interactions. **b** The four most common biological phenotypes associated with CSNK2 in cancer, categorised by cancer hallmark [59] were: apoptosis (A), proliferation (P), cell cycle control (C) and invasion/metastasis (I). These four phenotypes were paired to the pathways from **a** if the pathway was an established regulator of this biological behaviour.

Mechanistically, five cancer-specific CSNK2 phosphotargets were identified in at least four or more cancer types: AKT, STAT3, RELA (NFkB), PTEN and TP53 (Table 1 with corresponding references in Table S1, Appendix 2; Fig. 1a). Each of these targets regulate processes directly related to hallmark oncology phenotypes [59]. The most heavily cited was AKT, identified in 17 of these cancers. Discovered 27 years ago, AKT is known to play a widespread role in cell survival, proliferation, metabolism, and growth, among others. As such, its functionality is exploited in cancer, demonstrated in part by the aberrant activation of AKT in 77% of all metastatic melanoma lesions [60] and 32% of all colorectal cancers [61]. Although the activity of AKT is influenced by many factors, CSNK2 exhibits site-specific phosphorylation of S129 that has been shown to positively regulate AKT’s catalytic activity [28]. This specificity is important since other regulators of AKT, such as serine-threonine kinase GSK3A (a member of the same kinase family as CSNK2), phosphorylates T312 of AKT leading to activity attenuation instead of stimulation [62]. Thus, despite the pleiotropy of CSNK2, it demonstrates a specificity that is likely very biologically significant.

At the phenotypic level, the most commonly cited behaviours influenced by CSNK2 in cancer were evasion of apoptosis (apoptosis), enhancement of proliferation (proliferation), enhancement of invasion/metastasis (invasion and metastasis), and cell cycle control (Table 1 with references in Table S1 and Appendix 2; Fig. 1b). Each of these were reported in 11 or more cancers. It was thus no surprise that they correlated heavily with commonly cited CSNK2-targets mentioned above. For example, AKT, PTEN and NFkB are known to regulate components of all four of these phenotypes. Although these were the most cited, virtually all phenotypic hallmarks of cancer were found to be influenced by CSNK2 in at least one context. Such examples included modulation of glucose metabolism in favor of maximal glycolytic capacity in colorectal cancer cells [63], and the upregulation of an important DNA-repair mechanism in gastric cancer cells with cisplatin-induced DNA damage [64].

In summary, it is clear that distinct biological patterns exist for CSNK2 in cancer. These patterns span a broad spectrum of biology, from modulating gene transcription to phenotypic behaviours. Although widespread patterns did exist, the role in CSNK2 in oncogenesis is complex and variation was present throughout. Nonetheless, this repository of information offers exciting insights using systematically collected data that is specific to CSNK2 biology in cancer.

## Translational applications of CSNK2 in cancer

As outlined in the previous section, CSNK2 is widely dysregulated in cancer and is well positioned to be a high-yield clinical target. As a prelude to clinical trials, numerous studies have been conducted on CSNK2 to investigate the efficacy of targeting it in vivo. Compiled in Table 2 and Table S1 (corresponding references in Appendix 2) are representative experiments from systematically collected data of xenograft experiments that employed various methods of CSNK2 inhibition or knockdown. The principal conclusion from these experiments was that, irrespective of methodology, downregulation of CSNK2 almost always yielded positive outcomes. For example, intra-peritoneal injection of P15-Tat (i.e. CIGB-300) in female nude mice engrafted with H-125 human NSCLC cells resulted in an improvement in survival from 24 to 41 days compared to control (*p* = 0.0002) [65]. In another model, nude mice were engrafted with FaDu cells to create a hypopharyngeal SCC xenograft. After 35 days of treatment with RNAi-CSNK2 through tail injection, tumour volumes were ~75 mm^3^ compared to 1075 mm^3^ in the control (*p* < 0.0001) [66]. Examples such as these provide a strong foundation on which to further the clinical study of CSNK2 inhibition.

The pre-clinical and clinical efficacy of CSNK2 inhibition or knockdown is likely context dependent, however. An excellent example of this was shown in a prostate cancer mouse xenograft model, where CSNK2A1 siRNA was delivered via a nanoparticle delivery system in PC3-LN4 engrafted mice, leading to a >50% reduction in tumour volume at 10 days after the start of treatment (*p* = 0.005) [67]. In this same experiment, however, castrate-resistant prostate cancer cells (22Rv1) did not show any significant reduction in tumour volume. This study offered evidence that the biological background of CSNK2 inhibition/knockdown may be crucial in defining treatment efficacy. Coupled with several other examples in Table 2, the efficacy of CSNK2 inhibition can be strongly context dependent and must be accounted for when selecting the highest-yield clinical targets for further investigation.

Overall, CSNK2 has demonstrated itself to be a promising pre-clinical target in several cancers. Due in part to these findings, several preliminary experiments utilising CSNK2 inhibition in humans have been published to date. In a preliminary study of 31 women with micro-invasive or pre-invasive cervical cancer, intra-lesional injections of increasing doses of CIGB-300 over 5 days led to 75% of patients experiencing a significant visible lesion reduction, and 19% experiencing a full histologic regression [68]. Furthermore, no maximum-tolerated dose or dose-limiting toxicity was seen. In yet another example, a visually striking case was shown in a compassionate treatment program for a patient with chemo-radio-refractory metastatic germinoma to the spine. CIGB-300 was injected intra-lesionally, with a dramatic reduction in tumour volume after 7 days alongside an accompanying relief of symptoms, which was maintained for at least 1 year during the study’s follow-up [69].

Formalised phase I and II clinical trials are underway to explore anti-CSNK2 therapies in the USA. Among them is NCT0212828, a phase I/II trial investigating the safety and preliminary efficacy of CX4945 in combination with cisplatin and gemcitabine in unresectable or metastatic cholangiocarcinoma. Anticipated study completion date is August 2021. Several other studies have been initiated, but not completed or their data remains unavailable publicly (Table 3). Further, no trials on CSNK2 were found through database searches of the EU Clinical Trials Register, Cancer Research UK Registry or Canadian Clinical Trials database. As such, there still remains a significant need to improve the knowledge around, and access to, CSNK2 inhibitor or knockdown therapies.

**Table 3:** Summary of oncology clinical trials identified within the NIH database that selectively targeted CSNK2.

Cancer type	Study type	Population cohort	Intervention	Results	Reference
Cholangiocarcinoma	Phase I/II Clinical Trial	Unresectable or metastatic cholangiocarcinoma	Assessment of maximum tolerated dose CX4945, then administered with standard of care gemcitabine + cisplatin	Anticipated study completion August 2021.	NCT02128282
Multiple myeloma	Phase I Clinical Trial	Relapsed or refractory MM after at least 2 lines of therapy	CX4945 QID—dose escalation study	Study completed September 2011.	NCT01199718
Cervical	Phase II Clinical Trial	Local application of CIGB-300 to cervical adenocarcinoma or SCC	CIGB-300 15 mg, 35 mg, 75 mg locally applied	Study completed August 2016.	NCT01639625
Multiple sites	Phase I Clinical Trial	Dose escalation study for breast cancer, multiple myeloma, Castleman’s, advanced solid tumours	CX4945 BID or QID—dose escalation study	Study not completed.	NCT00891280

Cancer type, study phase, population, intervention, results and NCT number of the studies were listed. No oncology clinical trials targeting CSNK2 were identified in the EU Clinical Trials Register, Cancer Research UK Registry or Canadian Clinical Trials databases.

In addition to the therapeutic applications of CSNK2 modulation, there exist other areas in which this kinase has demonstrated clinical utility, namely that of diagnosis and prognostication. CSNK2 was found to hold prognostic significance in 14 of the cancers in this review (Table S1 with references in Appendix 2). Several were found to have diagnostic utility as well. For example, in clear cell renal cell carcinoma (RCC), Kaplan–Meier analysis of CSNK2A1 mRNA levels revealed a strong inverse correlation with overall survival (*p* < 0.01), TNM stage (*p* = 0.02) and metastasis (*p* = 0.003) [70]. In cholangiocarcinoma cells, Kaplan–Meier analysis of CSNK2B protein levels were inversely correlated with overall survival (*p* = 0.003) [71]. From a diagnostic perspective, a screen of sera from normal and ovarian cancer patients revealed that immunoglobulins to CSNK2 were present only in sera from cancer patients [72]. Identification of CSNK2-specific immunoglobulins may prove to be a useful cancer diagnostic or screening tool with appropriate validation. Such studies reveal much promise in the utility of CSNK2 in diagnosis and prognostication.

In summary, CSNK2 holds significant clinical potential. Evidenced primarily through xenograft mouse models, CSNK2 inhibition or knockdown consistently demonstrates positive outcomes. This is strongly supportive of the pathophysiologic role it plays in oncogenesis. By modulating cell behaviours such as apoptosis, the epithelial mesenchymal transition (EMT), drug efflux, DNA-damage repair mechanisms, and more [73], CSNK2 is positioned to be a high-yield target for clinical exploration and application. Further study is clearly needed to better characterise its full therapeutic and diagnostic/prognostic value.

## Additional considerations

Over recent years, increasingly selective CSNK2 inhibitors have been used to improve the accuracy of scientific and pre-clinical studies such as CX4945 and CIGB-300. However, emerging data suggests that the use of these conventionally selective inhibitors may be less specific than previously thought. In a recent paper published by Wells et al. 2021 [74], a small library of highly selective pyrazolopyrimidine-based inhibitors significantly out-performed several conventional CSNK2 inhibitors in their selectivity in vitro. One of their highly selective compounds did not demonstrate anti-proliferative effects in the majority of cell lines tested, contrasting the widely held finding that CSNK2 is a universal driver of proliferation. As such, off-target effects of conventional inhibitors may have influenced results that were previously dependent on this method of CSNK2 inhibition, and must be considered when interpreting study outcomes. However, the use of conventional CSNK2 inhibitors as well as the targeted knockdown of CSNK2 in in vivo experiments have both demonstrated positive results in the xenograft setting (Table 2, with corresponding references in Appendix 2). Thus, the mechanisms by which small-molecule kinase inhibition and knockdown of CSNK2 may differ and must be accounted for when reconciling its biology, but they both harbor significant therapeutic potential.

CSNK2 has been identified as an excellent target for pre-clinical and clinical research, but the optimal application of its inhibition or knockdown remains largely unknown. One strategy that may maximise its potential is through co-administration with existing therapies. Doing so may facilitate increased anti-cancer efficacy, improved drug side effect tolerability at lower doses, and decreased resistance to chemotherapeutics. Multi-drug regimens that target pathways serially or in parallel to treat cancer have led to improved outcomes in several major clinical trials to date, such as melanoma [75, 76] and HER2+ metastatic breast cancer [77]. This strategy has already demonstrated efficacy in some xenograft models that employed CSNK2 inhibition [78]. Moreover, drawing on the findings of Wells and colleagues [74] that propose a narrower phenotypic spectrum of CSNK2 activity than previously thought, inhibition of CSNK2 may have a lower risk of cytotoxic side effects than may have been initially envisaged based on earlier conclusions that CSNK2 is essential for viability. Thus, CSNK2 remains a promising clinical target with boundless opportunity for exploration given the limited research conducted at this level to date.

In the era of PD-L1 and PD-1 inhibitors, a special note is made of the involvement of CSNK2 in this pathway. T-cells are an important component of the immune system’s ability to target and destroy cancer cells, within which the T-cell receptor (TCR) pathway plays a crucial role in its activation. By contrast, the PD-1/PD-L1 cascade helps to dampen T-cell activation, which cancer cells can take advantage of to evade host destruction. This mechanism begins with the recruitment of SHP phosphatase to PD-1 after its phosphorylation of several important cytoplasmic immunoreceptor motifs by Src kinases upon receptor stimulation [79]. SHP then dephosphorylates T-cell receptor proximal signaling components including CSNK2, PI3K/AKT, PTEN and RAS/MEK/ERK. As a result, T-cell proliferation, survival and cytokine production are inhibited, leading to lymphocyte exhaustion. In a simultaneous fashion, PD-1 also inhibits the stabilising effects of CSNK2-derived phosphorylation of PTEN, leading to downregulation of the PI3K/AKT pathway. Since the stimulation of PI3K/AKT and RAS/MEK/ERK pathways are co-required for T-cell activation, this effect further reduces T-cell activation [80]. Thus, the PD-1 pathway has a significant influence on CSNK2 and its effectors.

The net effect of systemic inhibition of CSNK2 in a cancer patient on PD-L1 or PD-1 inhibitors remains unclear, however. Downregulation of CSNK2 using therapies such as CX4945 or CIGB-300 on T-cells may theoretically negate some of the desired effects of PD-1/PD-L1 inhibitors. However, their systemic administration would likely be balanced against its effects on both other types of immune cells and the cancer itself. CSNK2 is known to influence the immune system and has been shown to increase immune-mediated destruction of cancer cells in several studies [81–84]. As a whole, identifying which pathways CSNK2 are implicated in will help in the focused exploration of its pathogenesis in multiple cells types, and may improve the selection of optimal pre-clinical and clinical therapeutic strategies. The information contained within this review serves as one anchoring repository of such data.

Lastly, and from a technical standpoint, the data and conclusions from this publication did have limitations and potential bias. The number of citations for each cancer was variable, with small cell lung cancer, parathyroid cancer, Hodgkin’s lymphoma, anal cancer, uterine cancer, testicular cancer and vulvar cancer being excluded from this review due to their paucity of data from preliminary searches. Another limitation was the observation that large knowledge deficits in specific aspects of CSNK2 biology exist. Such areas included CSNK2 post-translational modifications, subunit expression variation patterns and upstream regulatory mechanisms. Filling these gaps may lead to exciting new insights into CSNK2 pathophysiology. Third and lastly, by virtue of this review being systematic, certain studies were not included at the expense of utilising an unbiased approach to data collection. Nonetheless this affords the data strength in reducing bias not often seen in prior reviews of this kinase.

## Conclusion

Over 500 kinases are encoded in the human genome, which have been implicated in virtually all aspects of cellular function [2]. Targeting kinases has led to significant advancements in cancer treatment, representing the second-most common family of druggable targets, surpassed only by GPCRs [9]. Given their constitutive activity, diverse phosphoproteome, and distinct phosphorylation consensus sequence, CSNK2 has many distinguishable features. In this systematic review of 24 hematologic and solid malignancies, CSNK2 was shown to have readily identifiable patterns of pathophysiologic behaviour, and its inhibition has proven promising in animal models and several pre-clinical studies.

CSNK2 mRNA transcript, protein, and activity levels were found to be regularly elevated in cancer cells, evidenced from both data in this systematic review as well as data from the TCGA and CPTAC (as compiled by UALCAN [57]). Of the cancer-associated CSNK2 phosphotargets, AKT, STAT3, RELA (NFkB), PTEN and p53 were identified in four or more cancers (Table 1 with references in Table S1 and Appendix 2; Fig. 1). The most commonly cited phenotypes associated with CSNK2 in cancer were apoptosis, proliferation, invasion and metastasis and cell cycle control (Table 1 with corresponding references in Table S1, Appendix 2). There was considerable overlap between the studied CSNK2 targets & associated pathways, allowing for reconciliation of biochemical and microscopic behaviours. These trends help consolidate our understanding CSNK2 pathophysiology in cancer, but also may yield key insights into targeted clinical applications of its inhibition, such as small-molecule drugs that target parallel or serial pathways that CSNK2 is known to be implicated within the setting of cancer.

In summary, CSNK2 has proven to be an exciting kinase with unique pathophysiology and promising pre-clinical/clinical applications. Exploration of the utility of CSNK2 inhibition will likely offer new treatment strategies across multiple hematologic and solid malignancies in the coming years. It is anticipated that advancing our understanding CSNK2 biology will synergise with clinical data to empower the design of targeted treatment strategies to advance treatment options for disease, enabled in part by data such as that presented within this review. In combination with parallel studies and expert advice, targeted experiments may help to drive the discovery of new applications for CSNK2 inhibition that will positively impact patient lives.

## Supplementary information

Table S1

Table S2

Supplementary Information

PRISMA Checklist

## Data Availability

All data presented in this review have been made available through the supplemental appendices and tables.
