# Supplementary material for: CSNK2 in cancer: pathophysiology and translational applications
Source: Br J Cancer. 2021 Nov 12;126(7):994–1003. doi: 10.1038/s41416-021-01616-2 (PMC8980014; doi:10.1038/s41416-021-01616-2)
Supplement: Supplementary file 1 — Table S1 [file 41416_2021_1616_MOESM1_ESM.docx]

| **Cancer Type** | **CSNK2A1**  **Protein** | **CSNK2A2**  **Protein** | **CSNK2B**  **Protein** | **CSNK2A1 Transcript** | **CSNK2A2**  **Transcript** | **CSNK2B**  **Transcript** | **CSNK2**  **Activity** | **CSNK2 Phosphotargets in Cancer** | **CSNK2 Oncogenic Phenotypes** |  | **CSNK2 as a Prognostic Marker** | ***In Vitro* Data** | **Xenograft/*In Vivo* Data** |  |
| --- | --- | --- | --- | --- | --- | --- | --- | --- | --- | --- | --- | --- | --- | --- |
| **Bladder** | IN (Shimada 2011, Zhang 2016) |  |  | IN (Zhang 2016) |  |  |  | AKT (Zhang 2016). AKT (Shimada 2011). HDAC2 (Jia 2016). | Proliferation, metabolism (Zhang 2016). Cell cycle (Shimada 2011). |  |  | Zhang 2016 | shRNA (Zhang 2016) |  |
| **Bone** | IN (Takahashi 2017) |  | IN (Takahashi 2017) |  |  |  |  | FGF1 (Skjerpen 2002). SHOX (Marchini 2006). PLEKHO1, CAPZA1 (Canton 2005). MDC1 (Melander 2008). OTUB1 (Herhaus 2015). | Proliferation, apoptosis (Takahashi 2017). Apoptosis (Di Maira 2007). Proliferation* (Litchfield 2001). Proliferation* (Vilk 1999). |  |  | Takahashi 2017. Di Maira 2007. | CX4945 (Takahasi 2017) |  |
| **Brain (GBM)** | IN (Dubois 2016, Nitta 2015, Dixit 2012) |  |  | IN (Nitta 2015, Zheng 2013, Dubois 2016) | IN (Dubois 2016) | IN (Dubois 2016) | IN (Dubois 2016) | PGK1 (Li 2018). MTOR, MAPK1 (Olsen 2012). CAST (Bassett 2018). CTNNB (Nitta 2015). AKT (Chen 2019). PTEN (Miller 2002). SIRT1 (Dixit 2012). IRF3, TBK1 (Pencheva 2017). RELA (Schaefer 2017). PPP2CA (Mandal 2014). RELA, AKT, STAT3, STAT5 (Zheng 2013). CTNNA (Ji 2009). | Cell cycle , proliferation (Chen 2019). Proliferation, evading growth suppression (Pucko 2019). Apoptosis (Guerra 2015). Proliferation (Nitta 2015). Invasion and migration, evading growth suppression, apoptosis (Zheng 2013). Apoptosis (Olsen 2012). Apoptosis, evading growth suppression (Dixit 2012). Apoptosis (Olsen 2010). |  |  | Dubois 2016. Dixit 2016. Ghildiyal 2015. Guerra 2015. | siRNA (Chou 2016). TBB (Dixit 2016). TBB (Chen 2019). CX4945 (Pencheva 2017). shRNA (Nitta 2015). CX4945 (Zheng 2013) |  |
| **Breast** | IN (Das 2016, Munstermann 1990) |  |  | IN (Kren 2015) | DEC (Kren 2015) | IN (Kren 2015) | IN (Romieu-Mourez 2001, Landesman-Bollag 2001) | AKT, CDKN1A, HIF1A (Siddiqui-Jain 2010). PTEN (Torres 2001). CCDC106 (Ning 2019). SIRT6 (Bae 2016). MYH2 (Dulyaninova 2007). FOXC2 (Golden 2015). ESR1 (Arnold 1994). ESR1 (Williams 2009). PGR (Hagan 2013, 2011). PGR (Zhang 1994). ESR1 (Arnold 1995). MAPK14, MAPK8 (Kim 2018). SIRT6 (Schmidt 2018). IKBKE (Eddy 2005). NFKBIA (Romieu-Mourez 2001). STAT3 (Zheng 2011). GPI (Kho 2014). STAT3 (Drygin 2011). SLC39A7 (Taylor 2012). HDAC2 (Sun 2002). SIX1 (Ford 2000). AKT (Park 2016). AKT (Das 2016). CTNNB (Yefi 2011). IGFBP3 (Pattison 1999). AKT, MTOR, RELA, STAT3 (Gray 2014). PRKAA2 (Jang 2020). PRKC (Borner 1989). | Apoptosis, cell cycle, angiogenesis, (Siddiqui-Jain 2010). Apoptosis (Ning 2019). Invasion and migration (Kim 2018). Cell cycle (Park 2016). Invasion and migration (Dulyaninova 2007). Apoptosis, invasion and migration (Romieu-Mourez 2002). Proliferation, invasion and migration (Das 2016). Proliferation (Williams 2015). Proliferation (Kren 2015). Invasion and migration (Deshiere 2013). Invasion and migration (Golden 2015). Cell cycle, evading growth suppression, invasion and migration (Gray 2014). Cell cycle, apoptosis (Li 2014). Cell cycle (Ford 2000). Proliferation, invasion and migration (Bae 2016). Apoptosis (Ravi 2002). Evading growth suppression (Tapia 2006). |  | Prognosis (Giusiano 2011). Survival (Bae 2016). Survival (Williams 2015). | Gray 2014. Yde 2007. Tapia 2006. Winska 2018. | CX4945 (Siddiqui-Jain 2010). RNAi (Trembley 2017). siRNA (Kren 2015). |  |
| **Cervical** |  |  |  |  |  |  |  | CCDC106 (Ning 2019). NPM1 (Perea 2011). | Apoptosis (Ning 2019). Apoptosis, cell cycle (Yamane 2005). Apoptosis (Hellwig 2010). |  | Prognosis (Chua 2017) | Perera 2014 | CIGB300 (Perera 2014). CIGB300 (Perea 2008) |  |
| **Cholangiocarcinoma** | IN (Kotawong 2016, Di Maira 2019) | IN (Di Maira 2019) | IN (Zhou 2013, Di Maira 2019) | IN (Di Maira 2019) | IN (Di Maira 2019) | IN (Di Maira 2019) | IN (Di Maira 2019) | AKT (Di Maira 2019). | Apoptosis, evading growth suppression (Lustri 2017). Proliferation, apoptosis (Lertsuwan 2018). Proliferation, invasion and migration, cell cycle (Di Maira 2019) |  | Survival (Zhou 2014). |  |  |  |
| **Colorectal** | IN (Zou 2011, Munstermann 1990, Liang 2007) |  |  |  |  |  |  | RIOK1 (Hong 2018). ECE1 (Niechi 2015). TP53 (Kang 2009). HSP90 (Kim 2015). DUSP4 (Lee 2011). EIF5 (Homma 2005). CASTOR1 (Silva-Pavez 2019). TOP1 (Roy 2014). CTNNB (Tapia 2006). PRKAA2 (Jang 2020) | Apoptosis (Ravi 2002). Proliferation, metabolism (Yang 2018). Proliferation, apoptosis (Yefi 2011). Proliferation, cell cycle, invasion and migration (Zou 2011). Proliferation (Homma 2005). Cell cycle (Kim 2012). Cell cycle (Lee 2014). Proliferation, apoptosis (Tapia 2006). Cell cycle, proliferation, apoptosis (Silva-Pavez 2019). Proliferation, invasion and migration (Zou 2011). |  | Survival (Hong 2018). Survival (Lin 2011) | Silva-Pavez 2019. Semaan 2018. Zou 2011. Farah 2003. |  |  |
| **Esophagus** |  |  | IN (Chen 2012) |  |  | IN (Chen 2012) |  | NCoR/SMRT (Yoo 2012). SNAI1, AKT (Ko 2012). BIRC5 (Juan 2011). | Invasion and migration (Yoo 2012). Apoptosis, invasion and migration (Ko 2012). Apoptosis (Juan 2011). |  |  | Chen 2012. |  |  |
| **Gastric** | IN (Bae 2015, Jiang 2019) |  | IN (Lin 2010) | IN (Jiang 2019) |  |  |  | RIOK1 (Hong 2018). PDCD5 (Choi 2015). CCAR2 (Bae 2015). ARC (Wang 2015). XRCC1 (Xu 2014). AKT, MTOR (Jiang 2019). | Invasion and migration, proliferation (Bae 2015). Invasion and migration (Lin 2010). DNA repair (Xu 2014). Apoptosis (Wang 2015). Invasion and migration (Lee 2014). Proliferation (Jung 2019). Invasion and migration (Jiang 2019). |  | Prognosis (Bae 2015). Prognosis (Lin 2010). | Kim 2018. Wang 2015. Chen 2007. |  |  |
| **Head & Neck** | IN (Brown 2010) | IN (Brown 2010) | IN (Brown 2010) |  |  |  | IN (Faust 2000, Faust 1996, Rydell 1990) | CTTN (Markwell 2019). IKBKB (Yu 2006). RELA (Brown 2010). AKT, RPS6, CDKN1A (Bian 2015). RELA (Unger 2014). TWIST (Su 2011). | Invasion and migration (Markwell 2019). Proliferation, evading growth suppression, invasion and migration, apoptosis (Brown 2010). Proliferation (Faust 2000). Cell cycle, proliferation (Bian 2015). Evading growth suppression (Lu 2014). Invasion and migration (Su 2011) |  | Survival (Gapany 1995). Prognosis (Faust 1996). | Brown 2010 | siRNA (Brown 2010). RNAi (Unger 2014). CX4945 (Bian 2015). |  |
| **Leukemia - AML** | IN (Quotti Tubi 2013, Kim 2007) |  |  | IN (Quotti Tubi 2017, Quotti Tubi 2013) |  | In (Quotti Tubi 2017) | IN (Kim 2007, Quotti Tubi 2013) | HOXA9 (Vijapurkar 2004). RELA, STAT3, FOXO3 (Quotti 2017). TP53 (Quotti Tubi 2013). AKT, PDK1, FOXO1, BAD (Kim 2007). SET (Arriazu 2020) | Proliferation, cell cycle (Quotti 2017). Apoptosis (Quotti Tubi 2013). Cell cycle, apoptosis (Kim 2007). Proliferation (Aasebo 2020). |  | Prognosis (Kim 2007) | Quotti Tubi 2017. Quotti Tubi 2013. Kim 2007. |  |  |
| **Leukemia - CLL** | IN (Martins 2010) |  | IN (Martins 2010) |  |  |  | IN (Martins 2010) | USP7 (Carra 2017). STAT3 (Rozovski 2017). PTEN, AKT (Martins 2014). PTEN (Martins 2011). PTEN, PRKCB, PRKCD (Martins 2010). PTEN, AKT (Shehata 2010). | Apoptosis, evading growth suppression (Martins 2010). Proliferation, evading growth suppression (Martins 2014). Apoptosis (Martins 2011). |  |  | Prins 2013. Martins 2011. Martins 2010. | CIGB300 (Martins 2013). CX4945 (Martins 2014). |  |
| **Liver** | IN (Kim 2014, Zhang 2015) |  |  | IN (Kim 2014, Zhang 2015, Sass 2011) | IN (Sass 2011) | IN (Kim 2014) | IN (Sass 2011, Cavin 2003) | AKT (Kim 2014). AKT (Zhang 2015). SEPTIN2 (Yu 2009). ASPH (Borgas 2015). TP53 (Hubert 2006). TOP2A (Chen 2011). IGFBP1 (Ankrapp 1996). NFKBIA (Cavin 2003). | Cell cycle (Kim 2014). Proliferation, invasion and migration, apoptosis (Zhang 2015). Invasion and migration (Wu 2014). Proliferation (Sass 2011). Apoptosis (Cavin 2003). Proliferation (Yu 2009). Avoiding immune destruction (Kim 2008). |  | Survival (Kim 2014). Survival (Zhang 2015). | Sass 2011. Kim 2008. | DMAT (Sass 2011) |  |
| **Lung (NSCLC)** | IN (Xie 2018) | IN (Liu 2016) |  |  |  |  | IN (Daya-Makin 1994) | BRMS1 (Liu 2016). PML (Scaglioni 2006). AKT, MTOR (So 2015). RPS3 (Yang 2013). PML (Yang 2017). CDC37, AKT (Stahl 2011). DVL (Jin 2019) | Invasion and metastasis (Liu 2016). DNA repair, apoptosis, cell cycle (Li 2017). Apoptosis (So 2015). |  | Survival (Liu 2016). Prognosis (Wang 2010). Survival (O-charoenrat 2004). | Li 2017. Yang 2017. So 2015. Cirigliano 2017. Bliesath 2012. Lin 2011. Gober 2019. Li 2019. | Tat-P15 (Perera 2008). |  |
| **Melanoma** | IN (Zhou 2016) |  |  |  |  |  | IN (Mitev 1994) | MAPK3/MAPK1, DUSP6 (Zhou 2016) |  |  |  | CX4945 synergistic with BRAF inhibitor (Parker 2014). |  |  |
| **Mesothelioma** | IN (Zhang 2014) |  |  | IN (Zhang 2014) |  |  |  |  |  |  |  | Zhang 2014 |  |  |
| **NHL - Follicular** | IN (Pizzi 2015) |  | IN (Pizzi 2015) |  |  |  |  |  |  |  |  |  |  |  |
| **NHL - DLBCL** |  |  |  |  |  |  |  | RELA, CDC37 (Pizzi 2015). AKT (Mandato 2018). | Proliferation, apoptosis (Mandato 2018) |  | Prognosis (Broseus 2016) |  |  |  |
| **Multiple Myeloma** | IN (Manni 2013, Piazza 2006) |  | IN (Manni 2013, Piazza 2006) |  |  |  | IN (Piazza 2006) | RELA, STAT3 (Manni 2014). STAT3, RELA (Manni 2013). STAT3 (Piazza 2006). TTI1, ETV7, AKT (Fernandez Saiz 2013). BRD4 (Wu 2013). | Apoptosis (Piazza 2006). Apoptosis (Manni 2014). Apoptosis, proliferation (Manni 2013). Apoptosis (Manni 2012). Apoptosis, evading growth suppression (Fernandez Saiz 2013) |  |  | Manni 2013. Piazza 2006. Manni 2014. |  |  |
| **Ovarian** | IN (Ma 2017) |  |  |  |  |  |  | BMI1 (Mustafi 2017). AKT, STAT3, HES1 (Kulbe 2016). GLI1 (Tang 2015). XRCC1, MDC1 (Siddiqui-Jain 2012). PTEN (Ali 2019) | Invasion and migration, apoptosis, proliferation (Ma 2017). Proliferation (Wang 2016). Apoptosis, proliferation (Kulbe 2016). |  | Survival (Ma 2017). Screening (Chatterjee 2006). | Kulbe 2016. Pathak 2015. Siddiqui-Jain 2012. | CX4945 (Chen 2019). CX4945 (Kulbe 2016). CX4945 with gemcitabine, carboplatin, cisplatin (Siddiqui-Jain 2012). |  |
| **Pancreatic** |  |  |  |  |  |  |  | AKT, CDKN1A, HIF1A (Siddiqui-Jain 2010). AKT, GSK3B (Kreutzer 2010). L1CAM (Chen 2010). | Apoptosis, cell cycle, anti-angiogenesis (Siddiqui-Jain 2010). Apoptosis, cell cycle (Hwang 2017). Invasion and migration (Chen 2010). Apoptosis (Hamacher 2007). Apoptosis (Giroux 2009). |  |  | Hwang 2017. Kreutzer 2010. Schaefer 2014. Guerra 2015. | CX4945 (Siddiqui-Jain 2010). siRNA (Giroux 2009) |  |
| **Prostate** | IN (Qaiser 2016) |  |  |  |  |  |  | RELA (Trembley 2019). HHEX (Siddiqui 2017). RELA, AR (Deng 2017). RELA (Gang 2015). AKT, STAT3, PML (Kalathur 2015). TP53 (Meenakshi 2014). PAWR (deThonel 2014). NKX3-1 (Li 2006). PAK1 (Shin 2013). PAK1 (Kim 2015). AKT (Pierre 2011). NCoR (Yoo 2013). PML, AKT (Chatterjee 2013). Vitamin D3 (Luo 2013). AKT, CDKN1A (Ryu 2012). AR (Yao 2012). AKT, RELA (Trembley 2011). NPM1 (Wang 2010). CDC25 (Schneider 2011). IGFBP3 (Cobb 2009). | Invasion and migration, proliferation (Siddiqui 2017). Apoptosis (Gang 2015). Apoptosis (de Thonel 2014). Invasion and migration, proliferation (Kim 2015). Invasion and migration (Yoo 2013). Proliferation (Kalathur 2014). Invasion and migration, proliferation, apoptosis (Chatterjee 2013). Proliferation (Trembley 2012). Apoptosis (Schneider 2011). Apoptosis (Cobb 2009). Apoptosis (Wang 2008, Wang 2006, Wang 2005, Wang 2005, Wang 2001). Apoptosis (Slaton 2004). Apoptosis (Ahmad 2007). Apoptosis, cell cycle (Pierre 2011). Apoptosis (Schneider 2009). Apoptosis (Ryu 2012). Evading growth suppression (Gotz 2012) |  | Prognosis (Shu 2016). Prognosis (Yoo 2013). Prognosis (Laramas 2007). | Pierre 2011. Koronkiewicz 2018. Deng 2017. Kalathur 2014. Ryu 2012. Schneider 2012. Gotz 2012. Yao 2012. Hessenauer 2011. Wang 2010. Schneider 2009. Schneider 2009. Trembley 2019. | CX4945 (Pierre 2011). Anti-sense RNA (Slaton 2004). Anti-sense RNA (Trembley 2011). RNAi (Trembley 2017). RNAi (Trembley 2014). RNAi (Ahmed 2016). TBB (Yoo 2013). DMAT (Trembley 2014). RNAi (Trembley 2019). |  |
| **Renal** | IN (Rabjerg 2017, Stalter 1994) |  | IN (Stalter 1994) | IN (Rabjerg 2017, Rabjerg 2016) | IN (Rabjerg 2017, Rabjerg 2016) | IN (Rabjerg 2017) | IN (Rabjerg 2017, Stalter 1994) | VHL (German 2016). CARD9 (Yang 2007). STAT1 (Timofeeva 2006). CTNNB (Yefi 2011). NFKBIA (Romieu-Mourez 2002). CTNNB (Tapia 2006). | Evading growth suppression, apoptosis (Tapia 2006). Invasion and migration (Rabjerg 2017). Invasion and migration (Vilardell 2017). Evading growth suppression (Yefi 2011). Avoiding immune suppression (Okada 2018). |  | Survival (Rabjerg 2016). Prognosis (Rabjerg 2017). Prognosis (Vilardell 2017). Survival (Chua 2017) | Romieu-Mourez 2002. Tapia 2006. |  |  |
| **Thyroid** | IN (Guo 2014) |  |  |  |  |  |  | AKT (Parker 2014) |  |  | Prognosis (Guo 2014). | CX4945 synergistic with vemurafenib or selumetinib (Parker 2014). |  |  |

**Table S1:** Summary of data from Table 1 and Table 2 with corresponding first author and year of publication notations, whose full citations are located in Appendix 2 of the Supplementary Information.
