# Supplementary material for: CSNK2 in cancer: pathophysiology and translational applications
Source: Br J Cancer. 2021 Nov 12;126(7):994–1003. doi: 10.1038/s41416-021-01616-2 (PMC8980014; doi:10.1038/s41416-021-01616-2)
Supplement: Supplementary file 2 — Table S2 [file 41416_2021_1616_MOESM2_ESM.docx]

| **Cancer Type** | **Concept 1 (CSNK2)** | **Concept 2 (Cancer and/or**  **histologic category, class)** | **Concept 3 (Location, subtype)** |
| --- | --- | --- | --- |
| Bladder | CSNK2 + CK2 + casein kinase 2 + casein kinase ii | Cancer + neoplasm + transitional cell + carcinoma | Bladder |
| Bone | CSNK2 + CK2 + casein kinase 2 + casein kinase ii | / | Osteosarcoma + osteogenic sarcoma |
| Brain | CSNK2 + CK2 + casein kinase 2 + casein kinase ii | Cancer + neoplasm + glioblastoma multiforme + GBM + glioblastoma | Brain |
| Breast | CSNK2 + CK2 + casein kinase 2 + casein kinase ii | Cancer + neoplasm + carcinoma + infiltrating ductal | Breast |
| Cervical | CSNK2 + CK2 + casein kinase 2 + casein kinase ii | Cancer + neoplasm + squamous cell + carcinoma | Cervical, cervix |
| Cholangiocarcinoma | CSNK2 + CK2 + casein kinase 2 + casein kinase ii | Cancer + neoplasm + adenocarcinoma | Bile duct, cholangiocarcinoma |
| Colorectal | CSNK2 + CK2 + casein kinase 2 + casein kinase ii | Cancer + neoplasm + adenocarcinoma | Colon, rectal, colorectal |
| Esophagus | CSNK2 + CK2 + casein kinase 2 + casein kinase ii | Cancer + neoplasm + adenocarcinoma | Esophagus, esophageal, oesophagus, oesophageal |
| Gastric | CSNK2 + CK2 + casein kinase 2 + casein kinase ii | Cancer + neoplasm + adenocarcinoma | Gastric, stomach |
| Head & neck | CSNK2 + CK2 + casein kinase 2 + casein kinase ii | Cancer + neoplasm + carcinoma | Oral, orophrayngeal, oropharynx, pharyngeal, pharynx, HNSCC |
| Leukemia - AML | CSNK2 + CK2 + casein kinase 2 + casein kinase ii | Leukemia, leukaemia | Acute myeloid, AML |
| Leukemia - CLL | CSNK2 + CK2 + casein kinase 2 + casein kinase ii | Leukemia, leukaemia | Chronic lymphocytic, CLL |
| Liver | CSNK2 + CK2 + casein kinase 2 + casein kinase ii | Cancer + neoplasm + carcinoma | Liver, hepatic, hepatocellular |
| Lung (NSCLC) | CSNK2 + CK2 + casein kinase 2 + casein kinase ii | Non-small-cell + non small cell + NSCLC | Lung, pulmonary |
| Melanoma | CSNK2 + CK2 + casein kinase 2 + casein kinase ii | / | Melanoma |
| Multiple Myeloma | CSNK2 + CK2 + casein kinase 2 + casein kinase ii | / | Myeloma, plasma cell myeloma, multiple myeloma |
| Mesothelioma | CSNK2 + CK2 + casein kinase 2 + casein kinase ii | Mesothelioma | Pleural, pleura, lung, pulmonary |
| NHL - Follicular | CSNK2 + CK2 + casein kinase 2 + casein kinase ii | NHL + non Hodgkin's + non Hodgkin + non-Hodgkin's + non-Hodgkin + lymphoma | Follicular |
| NHL - DLBCL | CSNK2 + CK2 + casein kinase 2 + casein kinase ii | NHL + non Hodgkin's + non Hodgkin + non-Hodgkin's + non-Hodgkin + lymphoma | DLBCL, DLBL, diffuse large B cell |
| Ovarian | CSNK2 + CK2 + casein kinase 2 + casein kinase ii | Cancer + neoplasm + epithelial + serous | Ovarian, ovary |
| Pancreatic | CSNK2 + CK2 + casein kinase 2 + casein kinase ii | Cancer + neoplasm + adenocarcinoma | Pancreatic, pancreas |
| Prostate | CSNK2 + CK2 + casein kinase 2 + casein kinase ii | Cancer + neoplasm + adenocarcinoma | Prostate, prostatic |
| Renal | CSNK2 + CK2 + casein kinase 2 + casein kinase ii | Cancer + neoplasm + carcinoma + adenocarcinoma + clear cell | Renal, kidney, nephro |
| Thyroid | CSNK2 + CK2 + casein kinase 2 + casein kinase ii | Cancer + neoplasm + follicular | Thyroid, thyroid gland |

**Table S2**: listing of terms used to construct each concept for this systematic review’s building block search strategy, as detailed in Appendix 1 of the Supplementary Information. Terms in each concept were combined using Boolean operators to generate final searches.
