# Supplementary material for: CSNK2 in cancer: pathophysiology and translational applications
Source: Br J Cancer. 2021 Nov 12;126(7):994–1003. doi: 10.1038/s41416-021-01616-2 (PMC8980014; doi:10.1038/s41416-021-01616-2)
Supplement: Supplementary file 3 — Supplementary Information [file 41416_2021_1616_MOESM3_ESM.docx]

**Supplementary Information:**

**Appendix 1: *Systematic Review Methodology***

A structured approach was applied to all data collection pertaining to CSNK2 pathophysiology. This protocol was designed specifically for this review using PRISMA methodology^133^. Data was specific to human cancer cell lines. Cancers chosen for this review represented a significant cross-section of adult solid and hematologic malignancies. For cancers with multiple major subtypes, the most common adult variants were included (ie. for Non-Hodgkin’s lymphoma, only DLBCL and follicular subtypes were assessed). All protein and enzyme names have been titled in accordance with HUGO Gene Nomenclature Committee (HGNC) standards.

Data extracted from each publication included information about: 1) CSNK2 subunit mRNA, protein, and kinase activity levels, 2) CSNK2 phosphorylation targets, 3) phenotypic changes attributable to changes in CSNK2 levels/activity, 4) *In vitro* and *in vivo* data related to CSNK2 levels/activity, 5) Data supporting prognostic significance of CSNK2 in cancer progression/survival. PubMed database was the study database utilized, inclusive of publications from all years. Systematically collected data contained herein are up to date as of May, 2020. Phenotypes were grouped as closely as possible to their respective hallmark of cancer^68^, guided by the work from Menyhart and colleagues^131^.

The Search Strategy employed was a building block strategy using three concepts: A) CSNK2, B) Cancer, C) Cancer type/primary cancer site. Each concept was searched individually, and thereafter combined using Boolean operators to generate final searches. A full listing of the terms used to construct each concept is found in Table S2. Study selection criteria have been listed below. Each abstract was manually evaluated to determine whether Screening Criteria were met. If insufficient information to make this decision was found in the abstract, the entire publication was reviewed. If the publication met Screening Criteria, the full publication was reviewed to see if it met Inclusion Criteria. If it did, all pertinent information from that publication was collected and catalogued (Table S1, with corresponding full citations in Appendix 2). A single reviewer facilitated all screening and data collection. No automated data collection tools were used at any point.

A total of 831 publications (duplicates included) were retrieved for initial review, with a total of 241 studies meeting both screening and inclusion criteria (duplicates excluded). Figure S1 depicts the corresponding PRISMA flow diagram. Note that any study containing data from *in vitro* or *in vivo* experiments using CK2 down-regulation must have done so using CSNK2-specific inhibitors (eg. CX4945, CIGB300, TBB, DMAT, etc…) or knockdown experiments. Data collected using from this systematic review methodology were summarized in Table 1, Table 2, and Table S1.

- Screening Criteria:
  - Must include information on CSNK2 protein kinase
  - Must be relevant to a specific cancer
  - Must include information relevant to human cells
- Inclusion Criteria:
  - CSNK2 mRNA, protein expression, and activity levels:
    - Must have demonstrated a change relative to non-cancer control(s)
  - CSNK2 phosphorylation site data must have demonstrated both:
    - A) Target phosphorylation by CSNK2 was either previously established or demonstrated within the publication itself
    - B) CSNK2 up/down-regulation was demonstrated to correlate with a change in the phosphorylation status of the target, OR substantial evidence was provided within the publication to support it as being differentially regulated in cancer
  - CSNK2-associated phenotypes:
    - CSNK2 up/down-regulation influenced cancer cell behavior *in vitro*
    - All phenotypes reported were GO biologic processes, grouped as one of the hallmarks of cancer^68^
  - CSNK2 *in vivo* data:
    - CK2 up/down-regulation influenced cancer cell behavior. Humans cells must have been the target tissue analyzed, but may have been demonstrated in xenograft models (any organism)


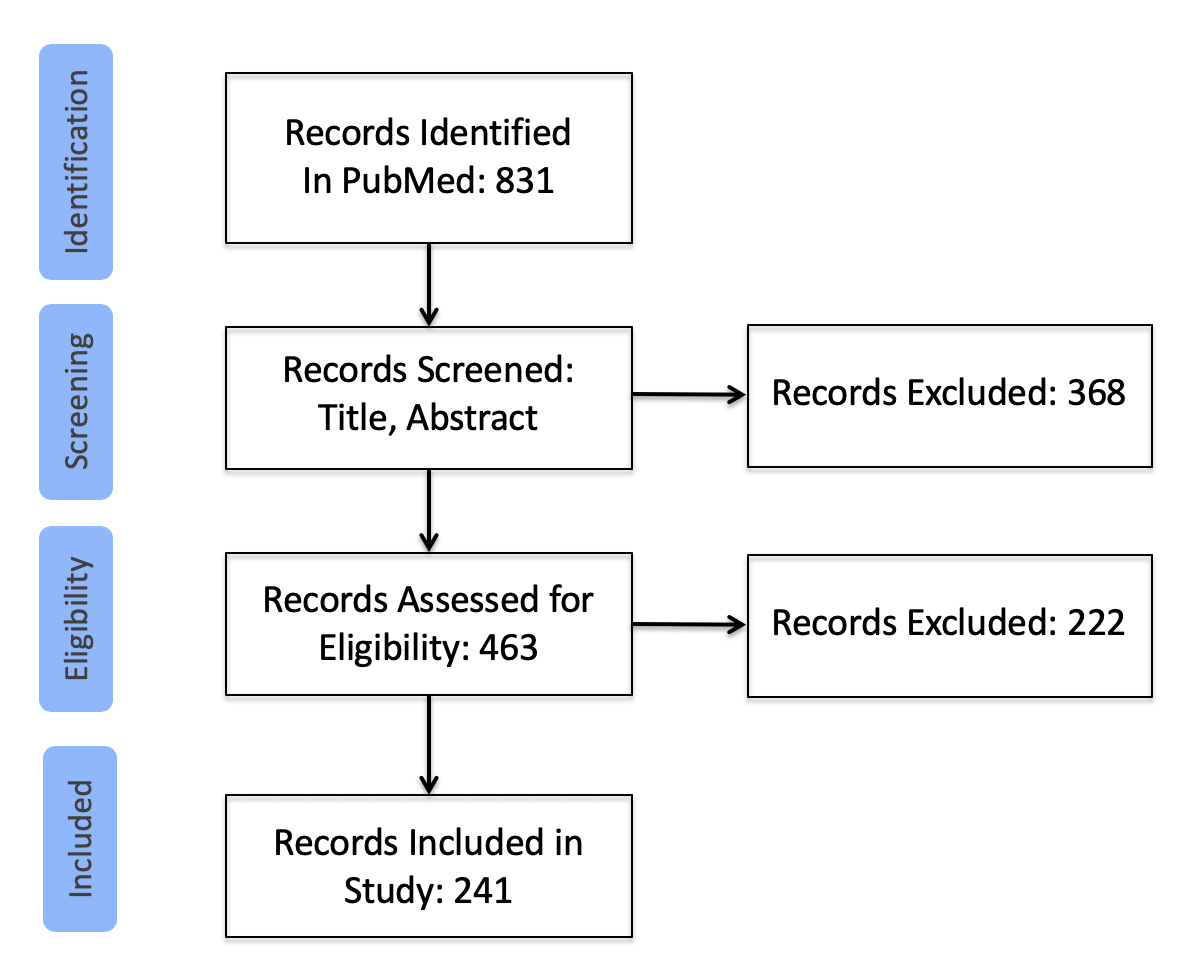


**Figure S1:** PRISMA flow diagram summarizing search results from the protocol described in the methods section. Building-block search strategy using Boolean combinations of concepts was used to search the PubMed database from all years. A total of 241 articles met inclusion criteria after screening, and were included in this study.

Of note, selected columns in Table 1 contained external data beyond what were collected in this systematic review. This external data encompassed mRNA transcript and protein levels from The Cancer Genome Atlas (TCGA)^245^ and CPTAC (Clinical Proteomic Tumor Analysis Consortium)^246^, respectively, as compiled by the UALCAN^247^ database. Transcript and protein levels were only considered different (increased or decreased) from normal cells if they were statistically significant. This data was incorporated to offer a direct comparator from large-scale high-throughput datasets.

**Appendix 2: *References for Table 1, Table S1, Table 2, Appendix 1***

1. Aasebø, E., Berven, F.S., Bartaula-Brevik. S., Stokowy, T., Hovland, R., Vaudel, M., et al. Proteome and Phosphoproteome Changes Associated with Prognosis in Acute Myeloid Leukemia. *Cancers* *(Basel)* **12**(3),709 (2020).

2. Ahmad, K.A., Harris, N.H., Johnson, A.D., Lindvall, H.C.N., Wang, G., Ahmed, K. Protein kinase CK2 modulates apoptosis induced by resveratrol and epigallocatechin-3-gallate in prostate cancer cells. *Mol Cancer Ther* **6**(3),1006–1012 (2007).

3. Ahmed, K., Kren, B.T., Abedin, M.J., Vogel, R.I., Shaughnessy, D.P., Nacusi, L., et al. CK2 targeted RNAi therapeutic delivered via malignant cell-directed tenfibgen nanocapsule: Dose and molecular mechanisms of response in xenograft prostate tumors. *Oncotarget* **7**(38),61789–805 (2016).

4. Ankrapp, D.P., Jones, J.I., Clemmons, D.R.. Characterization of insulin-like growth factor binding protein-1 kinases from human hepatoma cells. *J Cell Biochem* **60**(3),387–399 (1996).

5. Arnold, S.D., Obourn, J.D., Jaffe, H., Notides, A.C. Phosphorylation of the Human Estrogen Receptor by Mitogen-activated Protein Kinase and Casein Kinase II: Consequence on DNA binding. *Steroid Biochem Molec Biol* **55**(2),163–172 (1995).

6. Arnold, S.F., Obourn, J.D., Jaffe, H., Notides, A.C. Serine 167 is the major estradiol-induced phosphorylation site on the human estrogen receptor. *Mol Endocrinol* **8**(9),1208–1214 (1994).

7. Arriazu, E., Vicente, C., Pippa, R., Peris, I., Martínez-Balsalobre, E., García-Ramírez, P., et al. A new regulatory mechanism of protein phosphatase 2A activity via SET in acute myeloid leukemia. *Blood Cancer J* **10**(1),3 (2020).

8. Bae, J.S., Park, S.H., Jamiyandorj, U., Kim, K.M., Noh, S.J., Kim, J.R., et al. CK2α/CSNK2A1 Phosphorylates SIRT6 and Is Involved in the Progression of Breast Carcinoma and Predicts Shorter Survival of Diagnosed Patients. *Am J Pathol* **186**(12),3297–3315 (2016).

9. Bae, J.S., Park, S.H., Kim, K.M., Kwon, K.S., Kim, C.Y., Lee, H.K., et al. CK2α phosphorylates DBC1 and is involved in the progression of gastric carcinoma and predicts poor survival of gastric carcinoma patients. *Int J Cancer* **136**(4),797–809 (2015).

10. Banerjee, M.S., Chakraborty, P.K., Dwivedi, S.K.D., Ding, K., Moxley, K.M., Mukherjee, P., et al. BMI1, a new target of CK2α. *Mol Cancer* **16**(1),12–14 (2017).

11. Bassett, E.A., Palanichamy, K., Pearson, M., McElroy, J.P., Haque, S.J., Bell, E.H., et al. Calpastatin phosphorylation regulates radiation-induced calpain activity in glioblastoma. *Oncotarget* **9**(18),14597–14607 (2018).

12. Bhansali, M., Shemshedini, L.. COP9 subunits 4 and 5 target soluble guanylyl cyclase α1 and p53 in prostate cancer cells. *Mol Endocrinol* **28**(6),834–845 (2014).

13. Bian, Y., Han, J., Kannabiran, V., Mohan, S., Cheng, H., Friedman, J., et al. Mek inhibitor PD-0325901 overcomes resistance to CK2 inhibitor CX-4945 and exhibits anti-tumor activity in head and neck cancer. *Int J Biol Sci* **11**(4),411–422 (2015).

14. Bliesath, J., Huser, N., Omori, M., Bunag, D., Proffitt, C., Streiner, N., et al. Combined inhibition of EGFR and CK2 augments the attenuation of PI3K-Akt-mTOR signaling and the killing of cancer cells. *Cancer Lett* **322**(1),113–118 (2012).

15. Borgas, D., Gao, J-S., Tong, M., de la Monte, S. Regulation of Arpartyl-(Asparaginyl)-b-Hydroxylase Protein Expression and Function by Phosphorylation in Hepatocellular Carcinoma Cells. *JNSCI* **1**(4),e84 (2015).

16. Borner, C., Filipuzzi, I., Wartmann, M., Eppenberger, U., Fabbro, D. Biosynthesis and posttranslational modifications of protein kinase C in human breast cancer cells. *J Biol Chem* **264**(23),13902–13909 (1989).

17. Broséus, J., Chen, G., Hergalant, S., Ramstein, G., Mounier, N., Guéant, J.L., et al. Relapsed diffuse large B-cell lymphoma present different genomic profiles between early and late relapses. *Oncotarget* **7**(51),83987–84002 (2016).

18. Brown, M.S., Biallo, O.T., Hu, M., Ehsanian, R., Yang, X., Arun, P., et al. CK2 modulation of NF-kappaB, TP53 and the malignant phenotype in head and neck cancer by anti-CK2 oligonucleotides in vitro or in vivo via sub-50 nm nanocapsules Matthew. *Clin Cancer Res* **16**(8),2295–2307 (2010).

19. Canton, D.A., Olsten, M.E.K., Kim, K., Doherty-Kirby, A., Lajoie, G., Cooper, J.A., et al. The Pleckstrin Homology Domain-Containing Protein CKIP-1 Is Involved in Regulation of Cell Morphology and the Actin Cytoskeleton and Interaction with Actin Capping Protein. *Mol Cell Biol* **25**(9),3519–3534 (2005).

20. Carrà, G., Panuzzo, C., Torti, D., Parvis, G., Crivellaro, S., Familiari, U., et al. Therapeutic inhibition of USP7-PTEN network in chronic lymphocytic leukemia: A strategy to overcome TP53 mutated/ deleted clones. *Oncotarget* **8**(22),35508–35522 (2017).

21. Cavin, L.G., Romieu-Mourez, R., Panta, G.R., Sun, J., Factor, V.M., Thorgeirsson, S.S., et al. Inhibition of CK2 Activity by TGF-β1 Promotes IκB-α Protein Stabilization and Apoptosis of Immortalized Hepatocytes. *Hepatology* **38**(6),1540–1551 (2003).

22. Chatterjee, A., Chatterjee, U., Ghosh, M.K. Activation of protein kinase CK2 attenuates FOXO3a functioning in a PML-dependent manner: Implications in human prostate cancer. *Cell Death Dis* **4**(3),1–13 (2013).

23. Chatterjee, M., Mohapatra, S., Ionan, A., Bawa, G., Ali, R., Wang, X., et al. Diagnostic Markers of Ovarian Cancer by High-Throughput Antigen Cloning and Detection on Arrays. *Cancer Res* **66**(2),1181–1190 (2006).

24. Chen, B., Dong, Z., Cao, C., Wu, D., Huang, Z. Effect of casein kinase 2beta in esophageal carcinoma and its clinical significance. *J South Med Univ* **32**(10),1491–1494 (2012).

25. Chen, L.Y., Huang, R.L., Chan, M.W.Y., Yan, P.S., Huang, T.S., Wu, R.C., et al. TET1 reprograms the epithelial ovarian cancer epigenome and reveals casein kinase 2α as a therapeutic target. *J Pathol* **248**(3),363–376 (2019).

26. Chen, M.N., Lee, C-Y., Leland, H.A., Lin, G.Y., Montgomery, A.M., Silletti, S. Inside-Out Regulation of L1 Conformation, Integrin Binding, Proteolysis, and Concomitant Cell Migration. *Mol Biol Cell* **21**,1671–1685 (2010).

27. Chen, M-C., Chen, C-H., Chuang, H-C., Kulp, S.K., Teng, C-M., Chen, C-S. A novel mechanism by which histone deacetylase inhibitors facilitate topoisomerase IIα degradation in hepatocellular carcinoma cells. *Hepatology* **53**(1),148–159 (2011).

28. Chen, S.H., Lin, K.Y., Chang, C.C., Fang, C.L., Lin, C.P.. Aloe-emodin-induced apoptosis in human gastric carcinoma cells. *Food Chem Toxicol* **45**(11),2296–2303 (2007).

29. Chen, T., Chen, J., Zhu, Y., Li, Y., Wang, Y., Chen, H., et al. CD163, a novel therapeutic target, regulates the proliferation and stemness of glioma cells via casein kinase 2. *Oncogene* **38**(8),1183–1199 (2019).

30. Choi, H.K., Choi, Y., Park, E.S., Park, S.Y., Lee, S.H., Seo, J., et al. Programmed cell death 5 mediates HDAC3 decay to promote genotoxic stress response. *Nat Commun* **6**,7390 (2015).

31. Chou, S-T., Patil, R., Galstyan, A., Gangalum, P.R., Cavenee, W.K., Furnari, F.B., et al. Simultaneous blockade of interacting CK2 and EGFR pathways by tumor-targeting nanobioconjugates increases therapeutic efficacy against glioblastoma multiforme. *J Control Release* **244**(Part A),14–23 (2016).

32. Chua, M.M.J., Lee, M., Dominguez, I. Cancer-type dependent expression of CK2 transcripts. *PLoS ONE* **12**(12),1–44 (2017).

33. Cobb, L.J., Mehta, H., Cohen, P. Enhancing the apoptotic potential of insulin-like growth factor-binding protein-3 in prostate cancer by modulation of CK2 phosphorylation. *Mol Endocrinol* **23**(10),1624–1633 (2009).

34. Das, N., Datta, N., Chatterjee, U., Ghosh, M.K. Estrogen receptor alpha transcriptionally activates casein kinase 2 alpha: A pivotal regulator of promyelocytic leukaemia protein (PML) and AKT in oncogenesis. *Cell Signal* **28**(6),675–687 (2016).

35. Daya-Makin, M., Sanghera, J.S., Mogentale, T.L., Lipp, M., Parchomchuk, J., Hogg, J.C., et al. Activation of a Tumor-associated Protein Kinase (p40TAK) and Casein Kinase 2 in Human Squamous Cell Carcinomas and Adenocarcinomas of the Lung. *Cancer Res* **54**(8),2262–1168 (1994).

36. de Thonel, A., Hazoumé, A., Kochin, V., Isoniemi, K., Jego, G., Fourmaux, E., et al. Regulation of the proapoptotic functions of prostate apoptosis response-4 (Par-4) by casein kinase 2 in prostate cancer cells. *Cell Death Dis* **5**,e1016 (2014).

37. Deng, C., Chen, J., Guo, S., Wang, Y., Zhou, Q., Li, Z., et al. CX4945 suppresses the growth of castration-resistant prostate cancer cells by reducing AR-V7 expression. *World J Urol* **35**(8),1213–1221 (2017).

38. Deshiere, A., Duchemin-Pelletier, E., Spreux, E., Ciais, D., Combes, F., Vandenbrouck, Y., et al. Unbalanced expression of CK2 kinase subunits is sufficient to drive epithelial-to-mesenchymal transition by Snail1 induction. *Oncogene* **32**,1373–1383 (2013).

39. Di Maira, G., Brustolon, F., Bertacchini, J., Tosoni, K., Marmiroli, S., Pinna, L.A., et al. Pharmacological inhibition of protein kinase CK2 reverts the multidrug resistance phenotype of a CEM cell line characterized by high CK2 level. *Oncogene* **26**(48),6915–6926 (2007).

40. Di Maira, G., Gentilini, A., Pastore, M., Caligiuri, A., Piombanti, B., Raggi, C., et al. The protein kinase CK2 contributes to the malignant phenotype of cholangiocarcinoma cells. *Oncogenesis* **8**,61(2019).

41. Dixit, D., Sharma, V., Ghosh, S., Mehta, V.S., Sen, E.. Inhibition of Casein kinase-2 induces p53-dependent cell cycle arrest and sensitizes glioblastoma cells to tumor necrosis factor (TNFα)-induced apoptosis through SIRT1 inhibition. *Cell Death Dis* **3**(2),1–12 (2012).

42. Dixit, D., Ahmad, F., Ghildiyal, R., Joshi, S.D., Sen, E.. CK2 inhibition induced PDK4-AMPK axis regulates metabolic adaptation and survival responses in glioma. *Exp Cell Res* **344**(1),132–142 (2016).

43. Drygin, D., Ho, C.B., Omori, M., Bliesath, J., Proffitt, C., Rice, R., et al. Protein kinase CK2 modulates IL-6 expression in inflammatory breast cancer. *Biochem Biophys Res Commun* **415**(1),163–167 (2011).

44. Dubois, N., Willems, M., Nguyen-Khac, M-T., Kroonen, J., Goffart, N., Deprez, M., et al. Constitutive activation of casein kinase 2 in glioblastomas: Absence of class restriction and broad therapeutic potential. *Int J Oncol* **48**(6),2445–2452 (2016).

45. Duchemin-Pelletier, E., Baulard, M., Spreux, E., Prioux, M., Burute, M., Mograbi, B., et al. Stem cell-like properties of CK2β-down regulated mammary cells. *Cancers (Basel)* **9**(9),1–14 (2017).

46. Dulyaninova, N.G., House, R.P., Betapudi, V., Bresnick, A.R. Myosin-IIA Heavy-Chain Phosphorylation Regulates the Motility of MDA-MB-231 Carcinoma Cells. *Mol Biol Cell* **18**,3144–3155 (2007).

47. Eddy, S.F., Guo, S., Demicco, E.G., Romieu-Mourez, R., Landesman-Bollag, E., Seldin, D.C., et al. Inducible IκB kinase/IκB kinase ε expression is induced by CK2 and promotes aberrant nuclear factor-κB activation in breast cancer cells. *Cancer Res* **65**(24),11375–11383 (2005).

48. Farah, M., Parhar, K., Moussavi, M., Eivemark, S., Salh, B. 5,6-dichloro-ribifuranosylbenzimidazole- and apogenin- induced sensitization of colon cancer cells to TNF-alpha-mediated apoptosis. *Am J Physiol Gastrointest Liver* *Physiol* **285**(5),919–28 (2003).

49. Faust, R.A., Gapany, M., Tristani, P., Davis, A., Adams, G.L., Ahmed, K. Elevated protein kinase CK2 activity in chromatin of head and neck tumors: association with malignant transformation. *Cancer Lett* **101**(1),31–35 (1996).

50. Faust, R.A., Tawfic, S., Davis, A.T., Bubash, L.A., Ahmed, K. Antisense oligonucleotides against protein kinase CK2-α inhibit growth of squamous cell carcinoma of the head and neck in vitro. *Head Neck* **22**(4),341–346 (2000).

51. Fernandez-Saiz, V., Targosz, B-S., Lemeer, S., Eichner, R., Langer, C., Bullinger, L., et al. SCFFbxo9 and CK2 direct the cellular response to growth factor withdrawal via Tel2/Tti1 degradation and promote survival in multiple myeloma. *Nat Cell Biol* **15**(1),72–81 (2013).

52. Ford, H.L., Landesman-Bollag, E., Dacwag, C.S., Stukenberg, P.T., Pardee, A.B., Seldin, D.C. Cell cycle-regulated phosphorylation of the human SIX1 homeodomain protein. *J Biol Chem* **275**(29),22245–22254 (2000).

53. Gang, X., Wang, Y., Wang, Y., Zhao, Y., Ding, L., Zhao, J., et al. Suppression of casein kinase 2 sensitizes tumor cells to antitumor TRAIL therapy by regulating the phosphorylation and localization of p65 in prostate cancer. *Oncol Rep* **34**(3),1599–1604 (2015).

54. Gapany, M., Faust, R.A., Tawfic, S., Davis, A., Adams, G.L., Ahmed, K. Association of elevated protein kinase CK2 activity with aggressive behavior of squamous cell carcinoma of the head and neck. *Mol Med* **1**(6),659–666 (1995).

55. German, P., Bai, S., Liu, X-D., Sun, M., Zhou, L., Kalra, S., et al. Phosphorylation-dependent cleavage regulates von Hippel Lindau proteostasis and function. *Oncogene* **35**(38),4973–4980 (2016).

56. Ghildiyal, R., Sen, E. CK2 induced RIG-I drives metabolic adaptations in IFNγ-treated glioma cells. *Cytokine* **89**,219–228 (2017).

57. Giroux V, Iovanna J, Garcia S, Dagorn J-C. Combined inhibition of PAK7, MAP3K7, and CK2alpha kinases inhibits the growth of MiaPaCa2 pancreatic cancer cell xenografts. *Cancer Gene Ther* **16**(9),731–740 (2009).

58. Giusiano, S., Cochet, C., Filhol, O., Duchemin-Pelletier, E., Secq, V., Bonnier, P., et al. Protein kinase CK2α subunit over-expression correlates with metastatic risk in breast carcinomas: Quantitative immunohistochemistry in tissue microarrays. *Eur J Cancer* **47**(5),792–801 (2011).

59. Gober, M.K., Flight, R.M., Lambert, J., Moseley, H., Stromberg, A., Black, E.P. Deregulation of a Network of mRNA and miRNA Genes Reveals That CK2 and MEK Inhibitors May Synergize to Induce Apoptosis KRAS-Active NSCLC. *Cancer Inform* **18**,117693511984350 (2019).

60. Golden D, Cantley L. Casein Kinase 2 prevents mesenchymal transformation by maintaining Foxc2 in the cytoplasm. *Oncogene* **34**(36),4702–4712 (2015).

61. Götz, C., Gratz, A., Kucklaender, U., Jose, J. TF - A novel cell-permeable and selective inhibitor of human protein kinase CK2 induces apoptosis in the prostate cancer cell line LNCaP. *Biochim Biophys Acta* **1820**(7),970–977 (2012).

62. Gray, K.G., McFarland B.C., Rowse, L.R., Gibson, S.A., Benveniste, E.N. Therapeutic CK2 inhibition attenuates diverse prosurvival signaling cascades and decreases cell viability in human breast cancer cells. *Oncotarget* **5**(15),6484–6496 (2014).

63. Guerra, B, Fischer, M., Schaefer, S., Issinger, O.G. The kinase inhibitor D11 induces caspase-mediated cell death in cancer cells resistant to chemotherapeutic treatment. *J Exp Clin Cancer Res* **34**(1),1–14 (2015).

64. Guo, M., Liu, C., Qi, F.J., Zhang, X.M., Ren, L.L., Liu, Y.M., et al. Elevated expression of nuclear protein kinase CK2α as a poor prognosis indicator in lymph node cancerous metastases of human thyroid cancers. *Asian Pacific J Cancer Prev* **15**(17),7425–7432 (2014).

65. Hagan, C.R., Regan, T.M., Dressing, G.E., Lange, C.A. ck2-Dependent Phosphorylation of Progesterone Receptors (PR) on Ser81 Regulates PR-B Isoform-Specific Target Gene Expression in Breast Cancer Cells. *Mol Cell Biol* **31**(12),2439–2452 (2011).

66. Hagan, C.R., Knutson, T.P., Lange, C.A. A common docking domain in progesterone receptor-B links DUSP6 and CK2 signaling to proliferative transcriptional programs in breast cancer cells. *Nucleic Acids Res* **41**(19),8926–8942 (2013).

67. Hamacher, R., Saur, D., Fritsch, R., Reichert, M., Schmid, R.M., Schneider, G. Casein kinase II inhibition induces apoptosis in pancreatic cancer cells. *Oncol Rep* **18**(3),695–701 (2007).

68. Hanahan, D., Weinberg, R.A. Hallmarks of cancer: The next generation. *Cell* **144**(5),646–674 (2011)

69. Hellwig, C.T., Ludwig-Galezowska, A.H., Concannon, C.G., Litchfield, D.W., Prehn, J.H.M., Rehm, M. Activity of protein kinase CK2 uncouples Bid cleavage from caspase-8 activation. *J Cell Sci* **123**(9),1401–1406 (2010).

70. Herhaus, L., Perez-Oliva, A.B., Cozza, G., Gourlay, R., Weidlich, S., Campbell, D.G., et al. Casein kinase 2 (CK2) phosphorylates the deubiquitylase OTUB1 at Ser^16^ to trigger its nuclear localization. *Sci Signal* **8**(372),1–23 (2015).

71. Hessenauer, A., Schneider, C.C., Götz, C., Montenarh, M. CK2 inhibition induces apoptosis via the ER stress response. *Cell Signal* **23**(1),145–151 (2011).

72. Homma, M.K., Homma, Y. Regulatory role of CK2 during the progression of cell cycle. Mol *Cell Biochem* **274**(1–2),47–52 (2005).

73. Homma, M.K., Li, D., Krebs, E.G., Yuasa, Y., Homma, Y. Association and regulation of casein kinase 2 activity by adenomatous polyposis coli protein. *Proc Natl Acad Sci U S A* **99**(9),5959–5964 (2002).

74. Hong, X., Huang, H., Qiu, X., Ding, Z., Feng, X., Zhu, Y., et al. Targeting posttranslational modifications of RIOK1 inhibits the progression of colorectal and gastric cancers. *Elife* **7**,e29511 (2018).

75. Hubert, A., Paris, S., Piret, J.P., Ninane, N., Raes, M., Michiels, C. Casein kinase 2 inhibition decreases hypoxia-inducible factor-1 activity under hypoxia through elevated p53 protein level. *J Cell Sci* **119**(16),3351–3362 (2006).

76. Hwang, D.W., So, K.S., Kim, S.C., Park, K.M., Lee, Y.J., Kim, S.W., et al. Autophagy Induced by CX-4945, a Casein Kinase 2 Inhibitor, Enhances Apoptosis in Pancreatic Cancer Cell Lines. *Pancreas* **46**(4),575–581 (2017).

77. Jang, D.E., Song, J., Park, J-W., Yoon, S-H., Bae, Y-S.. Protein kinase CK2 activates Nrf2 via autophagic degradation of Keap1 and activation of AMPK in human cancer cells. *BMB Rep* **53**(5),272–277 (2020).

78. Ji, H., Wang, J., Nika, H., Hawke, D., Keezer, S., Ge, Q., et al. EGF-induced ERK activation promotes CK2-mediated disassociation of α-catenin from β-catenin and transactivation of β-catenin. *Mol Cell* **36**(4),547–559 (2009).

79. Jia, Z.M., Ai, X., Teng, J.F., Wang, Y.P., Wang, B.J., Zhang, X. p21 and CK2 interaction-mediated HDAC2 phosphorylation modulates KLF4 acetylation to regulate bladder cancer cell proliferation. *Tumor Biol* **37**(6),8293–8304 (2016).

80. Jiang, C., Ma, Z., Zhang, G., Yang, X., Du, Q., Wang, W. CSNK2A1 promotes gastric cancer invasion through the PI3K-AKT-mTOR signaling pathway. *Cancer Manag Res* **11**,10135–10143 (2019).

81. Jin, S.K., Ju, I.E., Cheong, J.W., Ae, J.C., Jin, K.L., Woo, I.Y., et al. Protein kinase CK2α as an unfavorable prognostic marker and novel therapeutic target in acute myeloid leukemia. *Clin Cancer Res* **13**(3),1019–28 (2007).

82. Juan, H.C., Tsai, H.T., Chang, P.H., Huang, C.Y.F., Hu, C.P., Wong, F.H. Insulin-like growth factor 1 mediates 5-fluorouracil chemoresistance in esophageal carcinoma cells through increasing survivin stability. *Apoptosis* **16**(2),174–83 (2011).

83. Jung, M., Park, K.H., Kim, H.M., Kim, T.S., Zhang, X., Park, S.M., et al. Inhibiting casein kinase 2 overcomes paclitaxel resistance in gastric cancer. *Gastric Cancer* **22**(6)1153-1163 (2019).

84. Kalathur, M., Toso, A., Chen, J., Revandkar, A., Danzer-Baltzer, C., Guccini, I., et al. A chemogenomic screening identifies CK2 as a target for pro-senescence therapy in PTEN-deficient tumours. *Nat Commun* **6,**7227 (2015).

85. Kang, J.Y., Kim, J.J., Jang, S.Y., Bae, Y.S. The p53-p21Cip1/WAF1 pathway is necessary for cellular senescence induced by the inhibition of protein kinase CKII in human colon cancer cells. *Mol Cells* **28**(5),489–494 (2009).

86. Kho, D.H., Zhang, T., Balan, V., Yi, W., Ha, S-W., Xie, Y., et al. Autocrine motility factor modulates EGF-mediated invasion signaling. *Cancer Res* **74**(8),2229–2237 (2014).

87. Kim, H.R., Kim, K., Lee, K.H., Kim, S.J., Kim, J. Inhibition of casein kinase 2 enhances the death ligand- and natural kiler cell-induced hepatocellular carcinoma cell death. *Clin Exp Immunol* **152**(2),336–344 (2008).

88. Kim, H.M., Jeong, I., Kim, H.J., Kang, S.K., Kwon, W.S., Kim, T.S., et al. Casein kinase 2 inhibitor, CX-4945, as a potential targeted anticancer agent in gastric cancer. *Anticancer Res* **38**(11),6171–6180 (2018).

89. Kim, H.S., Chang, Y.G., Bae, H.J., Eun, J.W., Shen, Q., Park, S.J., et al. Oncogenic potential of CK2α and its regulatory role in EGF-induced HDAC2 expression in human liver cancer. *FEBS J* **281**(3),851–861 (2014).

90. Kim, J.M., Noh, E.M., Song, H.K., You, Y.O., Jung, S.H., Kim, J.S., et al. Silencing of casein kinase 2 inhibits pkc-induced cell invasion by targeting MMP-9 in MCF-7 cells. *Mol Med Rep* **17**(6),8397–8402 (2018).

91. Kim, S.W., Hasanuzzaman, M., Cho, M., Heo, Y.R., Ryu, M.J., Ha, N.Y., et al. Casein kinase 2 (CK2)-mediated phosphorylation of Hsp90β as a novel mechanism of rifampin-induced MDR1 expression. *J Biol Chem* **290**(27),17029–17040 (2015).

92. Kim, S.Y., Lee, Y., Bae, Y. Biochemical and Biophysical Research Communications senescence by targeting a subunit of protein kinase CKII in human colorectal cancer cells. *Biochem Biophys Res Commun* **429**(3–4),173–179 (2012).

93. Kim, Y.B., Shin, Y.J., Roy, A., Kim, J.H. The role of the pleckstrin homology domain-containing protein CKIP-1 in activation of p21-activated kinase 1 (PAK1). *J Biol Chem* **290**(34),21076–21085 (2015).

94. Ko, H., Kim, S., Jin, C.H., Lee, E., Ham, S., Yook, J.I., et al. Protein kinase casein kinase 2-mediated upregulation of N-cadherin confers anoikis resistance on esophageal carcinoma cells. *Mol Cancer Res* **10**(8),1032–1038 (2012).

95. Koronkiewicz, M., Chilmonczyk, Z., Kazimerczuk, Z., Orzeszko, A. Deoxynucleosides with benzimidazoles as aglycone moiety are potent anticancer agents. *Eur J Pharmacol* **820**,146–155 (2018).

96. Kotawong, K., Thitapakorn, V., Roytrakul, S., Phaonakrop, N., Viyanant, V., Na-Bangchang, K. Plasma peptidome as a source of biomarkers for diagnosis of cholangiocarcinoma. *Asian Pacific J Cancer Prev* **17**(3),1163–1168 (2016).

97. Kren, B.T., Unger, G.M., Abedin, M.J., Vogel, R.I., Henzler, C.M., Ahmed, K, et al. Preclinical evaluation of cyclin dependent kinase 11 and casein kinase 2 survival kinases as RNA interference targets for triple negative breast cancer therapy. *Breast Cancer Res* **17**(1),1–21 (2015).

98. Kreutzer, J.N., Ruzzene, M., Guerra, B. Enhancing chemosensitivity to gemcitabine via RNA interference targeting the catalytic subunits of protein kinase CK2 in human pancreatic cancer cells. *BMC Cancer* **10**,440 (2010).

99. Kulbe, H., Iorio, F., Chakravarty, P., Milagre, C.S., Moore, R., Thompson, R.G., et al. Integrated transcriptomic and proteomic analysis identifies protein kinase CK2 as a key signaling node in an inflammatory cytokine network in ovarian cancer cells. *Oncotarget* **7**(13),15648–15661 (2016).

100. Landesman-Bollag, E., Romieu-Mourez, R., Song, D.H., Sonenshein, G.E., Cardiff, R.D., Seldin, D.C. Protein kinase CK2 in mammary gland tumorigenesis. *Oncogene* **20**(25),3247–3257 (2001).

101. Laramas, M., Pasquier, D., Filhol, O., Ringeisen, F., Descotes, J.L., Cochet, C. Nuclear localization of protein kinase CK2 catalytic subunit (CK2α) is associated with poor prognostic factors in human prostate cancer. *Eur J Cancer* **43**(5),928–934 (2007).

102. Lee, Y.S., Lee, D.Y., Yu, D.Y., Kim, S., Lee, Y.C. Helicobacter pylori induces cell migration and invasion through casein kinase 2 in gastric epithelial cells. *Helicobacter* **19**(6),465–475 (2014).

103. Lee, Y.H., Uhm, J.S., Yoon, S.H., Kang, J.Y., Kim, E.K., Kang, B.S., et al. The C-terminal domain of PLD2 participates in degradation of protein kinase CKII β subunit in human colorectal carcinoma cells. *BMB Rep* **44**(9),572–577 (2011).

104. Lertsuwan, J., Lertsuwan, K., Sawasdichai, A., Tasnawijitwong, N., Lee, K.Y., Kitchen, P., et al. CX-4945 induces methuosis in cholangiocarcinoma cell lines by a CK2-independent mechanism. *Cancers (Basel)* **10**(9),1–22 (2018).

105. Li, D., Chen, L., Hu, Z., Li, H., Li, J., Wei, C., et al. Alterations of microRNAs are associated with impaired growth of MCF-7 breast cancer cells induced by: Inhibition of casein kinase 2. *Int J Clin Exp Pathol* **7**(7),4008–4015 (2014).

106. Li, K., Zhou, F., Zhou, Y., Zhang, S., Li, Q., Li, Z., et al. Quinalizarin, a specific CK2 inhibitor, can reduce icotinib resistance in human lung adenocarcinoma cell lines. *Int J Mol Med* **44**(2),437–446 (2019).

107. Li, Q., Li, K., Yang, T., Zhang, S., Zhou, Y., Li, Z., et al. Association of protein kinase CK2 inhibition with cellular radiosensitivity of non-small cell lung cancer. *Sci Rep* **7**(1),16134 (2017).

108. Li, X., Guan, B., Maghami, S., Bieberich, C.J. NKX3.1 Is Regulated by Protein Kinase CK2 in Prostate Tumor Cells. *Mol Cell Biol* **26**(8),3008–3017 (2006).

109. Li, X., Qian, X., Jiang, H., Xia, Y., Zheng, Y., Li, J., et al. Nuclear PGK1 Alleviates ADP-Dependent Inhibition of CDC7 to Promote DNA Replication. *Mol Cell* **72**(4),650-660 (2018).

110. Liang, L., Qu, L., Ding, Y. Protein and mRNA characterization in human colorectal carcinoma cell lines with different metastatic potentials. *Cancer Invest* **25**(6),427–434 (2007).

111. Lin, K.Y., Fang, C.L., Chen, Y., Li, C.F., Chen, S.H., Kuo, C.Y., et al. Overexpression of nuclear protein kinase CK2 β subunit and prognosis in human gastric carcinoma. *Ann Surg Oncol* **17**(6),1695–1702 (2010).

112. Lin, Y.C., Hung, M.S., Lin, C-K., Li, J-M., Lee, K-D., Li, Y-C., et al. CK2 Inhibitors Enhance teh Radiosensitivity of Human Non-SMall Cell Lung Cancer Cells Through Inhibition of Stat3 Activation. *Cancer Biotherapy Radiopharm* **26**(3),381–388 (2011).

113. Litchfield, D.W., Bosc, D.G., Canton, D.A., Saulnier, R.B., Vilk, G., Zhang, C. Functional specialization of CK2 isoforms and characterization of isoform-specific binding partners. *Mol Cell Biochem* **227**(1–2),21–29 (2001).

114. Liu, Y., Amin, E.B., Mayo, M.W., Chudgar, N.P., Bucciarelli, P.R., Kadota, K., et al. CK2α’ Drives Lung Cancer Metastasis by Targeting BRMS1 Nuclear Export and Degradation. *Cancer Res* **76**(9),2675–2686 (2016).

115. Lu, H., Yan, C., Quan, X.X., Yang, X., Zhang, J., Bian, Y., et al. CK2 Phosphorylates and Inhibits TAp73 Tumor Suppressor Function to Promote Expression of Cancer Stem Cell Genes and Phenotype in Head and Neck Cancer. *Neoplasia* **16**(10),789–800 (2014).

116. Luo, W., Yu, W-D., Ma, Y., Chernov, M., Trump, D.L., Johnson, C.S. Inhibition of protein kinase CK2 reduces CYP24A1 expression and enhances 1,25-dihydroxyvitamin D3 anti-tumor activity in human prostate cancer cells. *Cancer Res* **73**(7),2289–2297 (2013).

117. Lustri, A.M., Di Matteo, S., Fraveto, A., Costantini, D., Cantafora, A., Napoletano, C., et al. TGF-β signaling is an effective target to impair survival and induce apoptosis of human cholangiocarcinoma cells: A study on human primary cell cultures. *PLoS One* **12**(9),e0183932 (2017).

118. Ma, Z., Wang, X., He, J., Xia, J., Li, Y. Increased expression of protein kinase CK2α correlates with poor patient prognosis in epithelial ovarian cancer. *PLoS On* **12**(3),e0174037 (2017).

119. Manda, T., Bhowmik, A., Chatterjee, A., Chatterjee, U., Chatterjee, S., Ghosh, M.K. Reduced phosphorylation of Stat3 at Ser-727 mediated by casein kinase 2 - Protein phosphatase 2A enhances Stat3 Tyr-705 induced tumorigenic potential of glioma cells. *Cell Signal* **26**(8),1725–1734 (2014).

120. Mandato, E., Nunes, S., Zaffino, F., Casellato, A., Macaccaro, P., Tubi, L., et al. CX-4945, a Selective Inhibitor of Casein Kinase 2, Synergizes with B cell Receptor Signaling Inhibitors in Inducing Diffuse Large B Cell Lymphoma Cell Death. *Curr Cancer Drug Targets* **18**(6),608–616 (2018).

121. Manni, S., Toscani, D., Mandato, E., Brancalion, A., Quotti Tubi, L., Macaccaro, P., et al. Bone marrow stromal cell-fueled multiple myeloma growth and osteoclastogenesis are sustained by protein kinase CK2. *Leukemia* **28**(10),2094–2097 (2014).

122. Manni, S., Brancalion, A., Mandato, E., Quotti Tubi, L., Colpo, A., Pizzi, M., et al. Protein Kinase CK2 Inhibition Down Modulates the NF-κB and STAT3 Survival Pathways, Enhances the Cellular Proteotoxic Stress and Synergistically Boosts the Cytotoxic Effect of Bortezomib on Multiple Myeloma and Mantle Cell Lymphoma Cells. *PLoS One* **8**(9),e75280 (2013).

123. Manni, S., Brancalion, A., Quotti Tubi, L., Colpo, A., Pavan, L., Cabrelle, A., et al. Protein kinase CK2 protects multiple myeloma cells from ER stress-induced apoptosis and from the cytotoxic effect of HSP90 inhibition through regulation of the unfolded protein response. *Clin Cancer Res* **18**(7),1888–1900 (2012).

124. Marchini, A., Daeffler, L., Marttila, T., Schneider, K.U., Blaschke, R.J., Schnölzer, M., et al. Phosphorylation on Ser106 modulates the cellular functions of the SHOX homeodomain protein. *J Mol Biol* **355**(3),590–603 (2006).

125. Markwell, S., Ammer, A., Interval, E., Allen, J., Papenberg, B., Hames, R., et al. Cortactin Phosphorylation by Casein Kinase 2 Regulates Actin- Related Protein 2/3 Complex Activity, Invadopodia Function and Tumor Cell Invasion. *Mol Cancer Res* **17**(4),987–1001 (2019).

126. Martins, L.R., Lúcio, P., Silva, M.C., Anderes, K.L., Gameiro, P., Silva, M.G., et al. Targeting CK2 overexpression and hyperactivation as a novel therapeutic tool in chronic lymphocytic leukemia. *Blood* **116**(15),2724–2731 (2010).

127. Martins, L.R., Lúcio, P., Silva, M.C., Gameiro, P., Silva, M.G., Barata, J.T. On CK2 regulation of chronic lymphocytic leukemia cell viability. *Mol Cell Biochem* **356**(1–2),51–55 (2011).

128. Martins, L.R., Perera, Y., Lúcio, P., Silva, M.G., Perea, S.E., Barata, J.T. Targeting chronic lymphocytic leukemia using CIGB-300, a clinical-stage CK2-specific cell-permeable peptide inhibitor. *Oncotarget* **5**(1),258–263 (2013).

129. Martins, L., Lucio, P., Melao, A., Antunes, I., Cardoso, B., Stansfield, R., et al. Activity of the clinical-stage CK2-specific inhibitor CX-4945 against chronic lymphocytic leukemia. *Leukemia* **28**,179–238 (2014).

130. Melander, F., Bekker-Jensen, S., Falck, J., Bartek, J., Mailand, N., Lukas, J. Phosphorylation of SDT repeats in the MDC1 N terminus triggers retention of NBS1 at the DNA damage-modified chromatin. *J Cell Biol* **181**(2),213–226 (2008).

131. Menyhárt, O., Harami-Papp, H., Sukumar, S., Schäfer, R., Magnani, L., de Barrios, O., et al. Guidelines for the selection of functional assays to evaluate the hallmarks of cancer. *Biochim Biophys Acta* **1866**(2),300–319 (2016).

132. Mitev, V., Miteva, L., Botev, I., Houdebine, L-M. Enhanced casein kinase II activity in metastatic melanoma. *J Dermatol Sci* **8**(1),45–49 (1994).

133. Moher, D., Liberati, A., Tetzlaff, J., Altman, D.G., Altman, D., Antes, G., et al. Preferred reporting items for systematic reviews and meta-analyses: The PRISMA statement. *PLoS Med* **6**(7),e1000097 (2009).

134. Munstermann, U., Fritz, G., Seitz, G., Tyiping, L., Schneider, H.R., Issinger, O‐G. Casein kinase II is elevated in solid human tumours and rapidly proliferating non‐neoplastic tissue. *Eur J Biochem* **189**(2),251–257 (1990).

135. Niechi, I., Silva, E., Cabello, P., Huerta, H., Carrasco, V., Villar, P., et al. Colon cancer cell invasion is promoted by protein kinase CK2 through increase of endothelin-converting enzyme-1c protein stability. *Oncotarget* **6**(40),42749–42760 (2015).

136. Ning, Y., Wang, C., Liu, X., Du, Y., Liu, S., Liu, K., et al. CK2-mediated CCDC106 phosphorylation is required for p53 degradation in cancer progression. *J Exp Clin Cancer Res* **38**(1),1–13 (2019).

137. Nitta, R.T., Gholamin, S., Feroze, A., Agarwal, M., Cheshier, S.H., Mitra, S.S., et al. Casein Kinase 2α Regulates Glioblastoma Brain Tumor Initiating Cell Growth through the β-Catenin Pathway. *Oncogene* **34**(28),3688–3699 (2015).

138. O-Charoenrat, P., Rusch, V., Talbot, S.G., Sarkaria, I., Viale, A., Socci, N., et al. Casein kinase II alpha subunit and C1-inhibitor are independent predictors of outcome in patients with squamous cell carcinoma of the lung. *Clin Cancer Res* **10**(17),5792–5803 (2004).

139. Okada, S.L., Simmons, R.M., Franke-Welch, S., Nguyen, T.H., Korman, A.J., Dillon, S.R., et al. Conditioned media from the renal cell carcinoma cell line 786.O drives human blood monocytes to a monocytic myeloid-derived suppressor cell phenotype. *Cell Immunol* **323**,49–58 (2018).

140. Olsen, BB., Issinger, O-G., Guerra, B. Regulation of DNA dependent protein kinase by protein kinase CK2 in human glioblastoma cells. *Oncogene* **29**,6016–6026 (2010).

141. Olsen BB, Svenstrup TH, Guerra B. Downregulation of protein kinase CK2 induces autophagic cell death through modulation of the mTOR and MAPK signaling pathways in human glioblastoma cells*. Int J Oncol* **41**(6),1967–1976 (2012).

142. Ortega, C.E., Seidner, Y., Dominguez, I. Mining CK2 in cancer. *PLoS One* **9**(12),e115609 (2014).

143. Park, S.Y., Bae, Y.S. Inactivation of the FoxO3a transcription factor is associated with the production of reactive oxygen species during protein kinase CK2 downregulation-mediated senescence in human colon cancer and breast cancer cells. *Biochem Biophys Res Commun* **478**(1),18–24 (2016).

144. Parker, R., Clifton-Bligh, R., Molloy, M.P. Phosphoproteomics of MAPK inhibition in BRAF-mutated cells and a role for the lethal synergism of dual BRAF and CK2 inhibition. *Mol Cancer Ther* **13**(7),1894–1906 (2014).

145. Pathak, H.B., Zhou, Y., Sethi, G., Hirst, J., Schilder, R.J., Golemis, E.A., et al. A Synthetic Lethality Screen Using a Focused siRNA Library to Identify Sensitizers to Dasatinib Therapy for the Treatment of Epithelial Ovarian Cancer. *PLoS One*  **10**(12),e0144126 (2015).

146. Pattison, S.T., Fanayan, S., Martin, J.L. Insulin-like growth factor binding protein-3 is secreted as a phosphoprotein by human breast cancer cells. *Mol Cell Endocrinol* **156**(1–2),131–139 (1999).

147. Pencheva, N., de Gooijer, M.C., Vis, D.J., Wessels, L.F.A., Würdinger, T., van Tellingen, O., et al. Identification of a Druggable Pathway Controlling Glioblastoma Invasiveness. *Cell Rep* **20**(1),48–60 (2017).

148. Perea, S.E., Baladrón, I., Garcia, Y., Perera, Y., Lopez, A., Soriano, J.L., et al. CIGB-300, a synthetic peptide-based drug that targets the CK2 phosphoaceptor domain. Translational and clinical research. *Mol Cell Biochem* **356**,45–50 (2011).

149. Perea, S.E., Baladrón, I., Valenzuela, C., Perera, Y. CIGB-300: A peptide-based drug that impairs the Protein Kinase CK2-mediated phosphorylation*. Semin Oncol* **45**(1–2),58–67 (2018).

150. Perera, Y., Toro, N., Gorovaya, L., Fernandez-de-cossio, J., Farina, H., Perea, S. Synergistic interactions of the anti-casein kinase 2 CIGB-300 peptide and chemotherapeutic agents in lung and cervical preclinical cancer models. *Mol Clin Oncol* **2**(6),935–944 (2014).

151. Perera, Y., Farina, H.G., Hernández, I., Mendoza, O., Serrano, J.M., Reyes, O., et al. Systemic administration of a peptide that impairs the Protein Kinase (CK2) phosphorylation reduces solid tumor growth in mice. *Int J Cancer* **122**(1),57–62 (2008).

152. Piazza, F.A., Ruzzene, M., Gurrieri, C., Montini, B., Bonanni, L., Chioetto, G., et al. Multiple myeloma cell survival relies on high activity of protein kinase CK2. *Blood* **108**(5),1698–1707 (2006).

153. Pierre, F., Chua, P., O’Brien, S., Siddiqui-Jain, A., Bourbon, P., Haddach, M., et al. Pre-clinical characterization of CX-4945, a potent and selective small molecule inhibitor of CK2 for the treatment of cancer. *Mol Cell Biochem* **356**,37–43 (2011).

154. Pizzi, M., Piazza, F., Agostinelli, C., Fuligni, F., Benvenuti, P., Mandato, E., et al. Protein kinase CK2 is widely expressed in follicular, Burkitt and diffuse large B-cell lymphomas and propels malignant B-cell growth. *Oncotarget* **6**(9),6544–6552 (2015).

155. Prins, R., Burke, R., Tyner, J., Druker, B., Loriaux, M., Spurgeon, S. CX-4945, a selective inhibitor of casein kinase-2 (CK2), exhibits anti-tumor activity in hematologic malignancies including enhanced activity in chronic lymphocytic leukemia when combined with fludarabine and inhibitors of the B-cell receptor pathway. *Leukemia* **27**,2094–2096 (2013).

156. Pucko, E., Ostrowski, R.P., Matyja, E. Novel small molecule protein kinase CK2 inhibitors exert potent antitumor eff2ects on T98G and SEGA cells in vitro. *Folia Neuropathol* **57**(3),239–248 (2019).

157. Qaiser, F., Trembley, J.H., Sadiq, S., Muhammad, I., Younis, R., Hashmi, S.N., et al. Examination of CK2α and NF-κB p65 expression in human benign prostatic hyperplasia and prostate cancer tissues. *Mol Cell Biochem* **420**(1–2),43–51 (2016).

158. Quotti Tubi, L., Canovas Nunes, S., Brancalion, A., Doriguzzi Breatta, E., Manni, S., Mandato, E., et al. Protein kinase CK2 regulates AKT, NF-κB and STAT3 activation, stem cell viability and proliferation in acute myeloid leukemia. *Leukemia* **31**(2),292–300 (2017).

159. Quotti Tubi, L., Gurrieri, C., Brancalion, A., Bonaldi, L., Bertorelle, R., Manni, S., et al. Inhibition of protein kinase CK2 with the clinical-grade small ATP-competitive compound CX-4945 or by RNA interference unveils its role in acute myeloid leukemia cell survival, p53-dependent apoptosis and daunorubicin-induced cytotoxicity. *J Hematol Oncol* **6**(1),1–15 (2013).

160. Rabjerg, M., Bjerregaard, H., Halekoh, U., Jensen, B.L., Walter, S., Marcussen, N. Molecular characterization of clear cell renal cell carcinoma identifies CSNK2A1, SPP1 and DEFB1 as promising novel prognostic markers. *APMIS* **124**(5),372–383 (2016).

161. Rabjerg, M., Guerra, B., Oliván-Viguera, A., Mikkelsen, M.L.N., Köhler, R., Issinger, O.G., et al. Nuclear localization of the CK2a-subunit correlates with poor prognosis in clear cell renal cell carcinoma. *Oncotarget* **8**(1),1613–1627 (2017).

162. Ravi, R., Bedi, A. Sensitization of tumor cells to Apo2 ligand/TRAIL-induced apoptosis by inhibition of casein kinase II. *Cancer Res* **62**(15),4180–4185 (2002).

163. Romieu-Mourez, R., Landesman-Bollag, E., Seldin, D.C., Sonenshein, G.E. Protein kinase CK2 promotes aberrant activation of nuclear factor-κB, transformed phenotype, and survival of breast cancer cells. *Cancer Res* **62**(22),6770–6708 (2002).

164. Romieu-Mourez, R., Landesman-Bollag, E., Seldin, D.C., Traish, A.M., Mercurio, F., Sonenshein, G.E. Roles of ikk kinases and protein kinase ck2 in activation of nuclear factor-κb in breast cancer. *Cancer Res* **61**(9),3810–3818 (2001).

165. Roy, A., Tesauro, C., Frøhlich, R., Hede, M.S., Nielsen, M.J., Kjeldsen, E., et al. Decreased camptothecin sensitivity of the stem-cell-like fraction of Caco2 cells correlates with an altered phosphorylation pattern of topoisomerase I. *PLoS One* **9**(6),e99628 (2014).

166. Rozovski, U., Harris, D.M., Li, P., Liu, Z., Jain, P., Veletic, I., et al. Constitutive Phosphorylation of STAT3 by the CK2-BLNK-CD5 Complex. *Mol Cancer Res* **15**(5),610–618 (2017).

167. Rydell, E., Axelsson, K., Olofsson, J., Hellem, S. Protein kinase activities in neoplastic squamous epithelia and normal epithelia from the upper aero-digestive tract. *Cancer Biochem Biophys* **11**(3),1187–1194 (1990).

168. Ryu, B.J., Baek, S.H., Kim, J., Bae, S.J., Chang, S.Y., Heo, J.N., et al. Anti-androgen receptor activity of apoptotic CK2 inhibitor CX4945 in human prostate cancer LNCap cells. *Bioorganic Med Chem Lett* **22**(17),5470–5474 (2012).

169. Sass, G., Klinger, N., Sirma, H., Hashemolhosseini, S., Hellerbrand, C., Neureiter, D., et al. Inhibition of experimental HCC growth in mice by use of the kinase inhibitor DMAT. *Int J Oncol* **39**(2),433–442 (2011).

170. Scaglioni, P.P., Yung, T.M., Cai, L.F., Erdjument-Bromage, H., Kaufman, A.J., Singh, B., et al. A CK2-Dependent Mechanism for Degradation of the PML Tumor Suppressor. *Cell* **126**(2),269–283 (2006).

171. Schaefer, S., Kreutzer, J.N., Issinger, O.G., Guerra, B. Cytotoxic effects exerted by pentachlorophenol by targeting nodal pro-survival signaling pathways in human pancreatic cancer cells. *Toxicol Reports* **1**,1162–1174 (2014).

172. Schaefer, S., Svenstrup, T.H., Guerra, B. The small-molecule kinase inhibitor D11 counteracts 17-AAG-mediated up-regulation of HSP70 in brain cancer cells. *PLoS One* **12**(5),e0177706 (2017).

173. Schmidt, J.A., Danielson, K.G., Duffner, E.R., Radecki, S.G., Walker, G.T., Shelton, A., et al. Regulation of the oncogenic phenotype by the nuclear body protein ZC3H8. *BMC Cancer* **18**(1),1–15 (2018).

174. Schneider, C.C., Gotz, C., Hessenauer, A., Gunther, J., Kartarius, S., Montenarh, M. Down-regulation of CK2 activity result in a decrease in the level of cdc25C phosphatase in different prostate cancer cell lines. *Mol Cell Biochem* **356**,177–184 (2011).

175. Schneider, C.C., Hessenauer, A., Gotz, C., Montenarh, M. DMAT, an inhibitor of protein kinase CK2 induces reactive oxygen species and DNA double strand breaks. *Oncol Rep* **21**,1593–1597 (2009).

176. Schneider, C.C., Kartarius, S., Montenarh, M., Orzeszko, A., Kazimierczuk, Z. Modified tetrahalogenated benzimidazoles with CK2 inhibitory activity are active against human prostate cancer cells LNCaP in vitro. *Bioorganic Med Chem* **20**(14),4390–4396 (2012).

177. Semaan, C., Neilson, K.A., Brown, B., Molloy, M.P. Pharmacological inhibition of casein kinase 2 enhances the effectiveness of PI3K inhibition in colon cancer cells. *Anticancer Res* **38**(11),6195–6200 (2018).

178. Shehata, M., Schnabl, S., Demirtas, D., Hilgarth, M., Hubmann, R., Ponath, E., et al. Reconstitution of PTEN activity by CK2 inhibitors and interference with the PI3-K/Akt cascade counteract the antiapoptotic effect of human stromal cells in chronic lymphocytic leukemia. *Blood* **116**(14),2513–2521 (2010).

179. Shimada, K., Anai, S., Marco, D.A., Fujimoto, K., Konishi, N. Cyclooxygenase 2-dependent and independent activation of Akt through casein kinase 2 contributes to human bladder cancer cell survival. *BMC Urol* **11**,8 (2011).

180. Shin, Y.J., Kim, Y.B., Kim, J.H. Protein kinase CK2 phosphorylates and activates p21-activated kinase 1. *Mol Biol Cell* **24**(18),2990–2999 (2013).

181. Shu, X., Ye, Y., Gu, J., He, Y., Davis, J.W., Thompson, T.C., et al. Genetic variants of the Wnt signaling pathway as predictors of aggressive disease and reclassification in men with early stage prostate cancer on active surveillance. *Carcinogenesis* **37**(10),965–971 (2016).

182. Siddiqui-Jain, A., Bliesath, J., Macalino, D., Omori, M., Huser, N., Streiner, N., et al. CK2 inhibitor CX-4945 suppresses DNA repair response triggered by DNA-targeted anticancer drugs and augments efficacy: Mechanistic rationale for drug combination therapy. *Mol Cancer Ther* **11**(4),994–1005 (2012).

183. Siddiqui-Jain, A., Drygin, D., Streiner, N., Chua, P., Pierre, F., O’Brien, S.E., et al. CX-4945, an orally bioavailable selective inhibitor of protein kinase CK2, inhibits prosurvival and angiogenic signaling and exhibits antitumor efficacy. *Cancer Res* **70**(24),10288–10298 (2010).

184. Siddiqui, Y.H., Kershaw, R.M., Humphreys, E.H., Assis Junior, E.M., Chaudhri, S., Jayaraman, P.S., et al. CK2 abrogates the inhibitory effects of PRH/HHEX on prostate cancer cell migration and invasion and acts through PRH to control cell proliferation. *Oncogenesis* **6**(1),1–8 (2017).

185. Silva-Pavez, E., Villar, P., Trigo, C., Caamaño, E., Niechi, I., Pérez, P., et al. CK2 inhibition with silmitasertib promotes methuosis-like cell death associated to catastrophic massive vacuolization of colorectal cancer cells. *Cell Death Dis* **10**(2),73 (2019).

186. Skjerpen, C.S., Nilsen, T., Wesche, J., Olsnes, S. Binding of FGF-1 variants to protein kinase CK2 correlates with mitogenicity. *EMBO J* **21**(15),4058–4069 (2002).

187. Slaton, J.W., Unger, G.M., Sloper, D.T., Davis, A.T., Ahmed, K. Induction of apoptosis by antisense CK2 in human prostate cancer xenograft model. *Mol Cancer Res* **2**(12),712–721 (2004).

188. So, K.S., Rho, J.K., Choi, Y.J., Kim, S.Y., Choi, C.M., Chun, Y.J., et al. AKT/mTOR down-regulation by CX-4945, a CK2 inhibitor, promotes apoptosis in chemorefractory non-small cell lung cancer cells. *Anticancer Res* **35**(3),1537–1542 (2015).

189. Ståhl, S., Branca, R.M., Efazat, G., Ruzzene, M., Zhivotovsky, B., Lewensohn, R., et al. Phosphoproteomic profiling of NSCLC cells reveals that ephrin B3 regulates pro-survival signaling through akt1-mediated phosphorylation of the EphA2 receptor. *J Proteome Res* **10**(10),2566-2578 (2011).

190. Stalter, G., Siemer, S., Becht, E., Ziegler, M., Remberger, K., Issinger, O-G. Asymmetric Expression of Protein Kinase CK2 Subunits in Human Kidney Tumors. *Biochem Biophys Res Commun* **202**(1),141–147 (1994).

191. Su, Y.W., Xie, T.X., Sano, D., Myers, J.N. IL-6 stabilizes twist and enhances tumor cell motility in head and neck cancer cells through activation of casein kinase 2. *PLoS One* **6**(4),e19412 (2011).

192. Sun, J.M., Chen, H.Y., Moniwa, M., Litchfield, D.W., Seto, E., Davie, J.R. The transcriptional repressor Sp3 is associated with CK2-phosphorylated histone deacetylase 2. *J Biol Chem* **277**(39),35783–35786 (2002).

193. Takahashi, K., Setoguchi, T., Tsuru, A., Saithoh, Y., Nagano, S., Ishidou, Y., et al. Inhibition of casein kinase 2 prevents growth of human osteosarcoma. *Oncol Rep* **37**,1141–1147 (2017)

194. Tang, A-Q., Cao, X-C., Tian, L., He, L., Liu, F. Apigenin inhibits the self-renewal capacity of human ovarian cancer SKOV-3-derived sphere-forming cells. *Mol Med Rep* **11**,2221–2226 (2015).

195. Tapia, J.C., Torres, V.A., Rodriguez, D.A., Leyton, L., Quest, A.F. Casein kinase 2 (CK2) increases survivin expression via enhanced beta-catenin-T cell factor/lymphoid enhancer binding factor-dependent transcription. *Proc Natl Acad Sci U S A* **103**(41),15079–15084 (2006).

196. Taylor, K.M., Hiscox, S., Nicholson, R.I., Hogstrand, C., Kille, P. Cell biology: Protein kinase CK2 triggers cytosolic zinc signaling pathways by phosphorylation of zinc channel ZIP7. *Sci Signal* **5**(210),8–10 (2012).

197. Timofeeva, O.A., Plisov, S., Evseev, A.A., Peng, S., Jose-Kampfner, M., Lovvorn, H.N., et al. Serine-phosphorylated STAT1 is a prosurvival factor in Wilms’ tumor pathogenesis. *Oncogene* **25**(58),7555–7564 (2006).

198. Torres, J., Pulido, R. The tumor suppressor PTEN is phosphorylated by the protein kinase CK2 at its C terminus. Implications for PTEN stability to proteasome-mediated degradation. *J Biol Chem* **276**(2),993–998 (2001).

199. Trembley, J.H., Unger, G., Tobolt, D.K., Korman, V.L., Wang, G., Ahmad, K.A., et al. Systemic administration of antisense oligonucleotides simultaneously targeting CK2α and α′ subunits reduces orthotopic xenograft prostate tumors in mice. *Mol Cell Biochem* **356**(1-2),21–35 (2011).

200. Trembley, J.H., Kren, B.T., Abedin, M.J., Vogel, R.I., Cannon, C.M., Unger, G.M., et al. CK2 Molecular Targeting—Tumor Cell-Specific Delivery of RNAi in Various Models of Cancer. *Pharmaceuticals* **10**(1),4–7 (2017).

201. Trembley, J.H., Kren, B.T., Abedin, M.J., Shaughnessy, D.P., Li, Y., Dehm, S.M., et al. CK2 Pro-Survival Role in Prostate Cancer Is Mediated via Maintenance and Promotion of Androgen Receptor and NFκB p65 *Expression. Pharmaceuticals* **12**(2),89 (2019).

202. Trembley, J.H., Unger, G.M., Gomez, O.C., Abedin, M.J., Korman, V.L., Vogel, R.I., et al. Tenfibgen-DMAT Nanocapsule Delivers CK2 Inhibitor DMAT to Prostate Cancer Xenograft Tumors Causing Inhibition of Cell Proliferation. *Mol Cell Pharmacol* **6**(2),15–25 (2014).

203. Trembley, J.H., Unger, G.M., Korman, V.L., Abedin, M.J., Nacusi, L.P., Vogel, R.I., et al. Tenfibgen ligand nanoencapsulation delivers bi-functional anti-CK2 RNAi oligomer to key sites for prostate cancer targeting using human xenograft tumors in mice. *PLoS One* **9**(10),e109970 (2014).

204. Unger, G., Kren, B., Korman, V., Kimbrough, T., Vogel, R., Ondrey, F., et al. Mechanism and efficacy of sub-50 nm tenfibgen nanocapsules for cancer cell-directed delivery of anti-CK2 RNAi to primary and metastatic squamous cell carcinoma. *Mol Cancer Ther* **13**(8),2018–2029 (2014).

205. Vijapurkar, U., Fischbach, N., Shen, W., Brandts, C., Stokoe, D., Lawrence, H.J., et al. Protein Kinase C-Mediated Phosphorylation of the Leukemia-Associated HOXA9 Protein Impairs Its DNA Binding Ability and Induces Myeloid Differentiation. *Mol Cell Biol* **24**(9),3827–3837 (2004).

206. Vilardell, J., Alcaraz, E., Sarró, E., Trilla, E., Cuadros, T., Torres, I., et al. Under-expression of CK2β subunit in ccRCC represents a complementary biomarker of p-STAT3 Ser727 that correlates with patient survival. *Oncotarget* **9**(5),5736–5751 (2018).

207. Vilk, G., Saulnier, R.B., St. Pierre, R., Litchfield, D.W. Inducible expression of protein kinase CK2 in mammalian cells. Evidence for functional specialization of CK2 isoforms. *J Biol Chem* **274**(20),14406–14414 (1999).

208. Wang, F., Chang, JT-H., Kao, C.J., Huang, R.S. High expression of miR-532-5p, a tumor suppressor, leads to better prognosis in ovarian cancer both in vivo and in vitro. *Physiol Behav* **15**(5),1123–1131 (2016).

209. Wang, G., Ahmad, K.A., Ahmed, K. Modulation of death receptor-mediated apoptosis by CK2. *Mol Cell Biochem* **274**(1–2),201–205 (2005).

210. Wang, G., Ahmad, K.A., Ahmed, K. Role of protein kinase CK2 in the regulation of tumor necrosis factor-related apoptosis inducing ligand-induced apoptosis in prostate cancer cells. *Cancer Res* **66**(4),2242–2249 (2006).

211. Wang, G., Pan, Y., Ahmad, K.A., Ahmed, K. Protein B23/Nucleophosmin/Numatrin nuclear dynamics in Relation to Protein Kinase CK2 and Apoptotic Activity in Prostate Cells. *Biochemistry* **49**(18),3842–3852 (2010).

212. Wang, H., Davis, A., Yu, S., Ahmed, K. Response of cancer cells to molecular interruption of the CK2 signal. *Mol Cell Biochem* **227**(1–2),167–174 (2001).

213. Wang, H., Yu, S., Davis, A.T., Ahmed, K. Cell cycle dependent regulation of protein kinase CK2 signaling to the nuclear matrix. *J Cell Biochem* **88**(4),812–822 (2003).

214. Wang, J., Feng, C., He, Y., Ding, W., Sheng, J., Arshad, M., et al. Phosphorylation of apoptosis repressor with caspase recruitment domain by protein kinase CK2 contributes to chemotherapy resistance by inhibiting doxorubicin induced apoptosis. *Oncotarget* **6**(29),27700–27713 (2015).

215. Wang, Z., Liu, H., Liu, B., Ma, W., Xue, X., Chen, J., et al. Gene expression levels of CSNK1A1 and AAC-11, but not NME1, in tumor tissues as prognostic factors in NSCLC patients. *Case Reports Clin Pract Rev* **16**(8),357–364 (2010).

216. Williams, C.C., Basu, A., El-Gharbawy, A., Carrier, L.M., Smith, C.L., Rowan, B.G. Identification of four novel phosphorylation sites in estrogen receptor α: Impact on receptor-dependent gene expression and phosphorylation by protein kinase CK2. *BMC Biochem* **10**(1),1–18 (2009).

217. Williams, M.D., Nguyen, T., Carriere, P.P., Tilghman, S.L., Williams, C. Protein kinase CK2 expression predicts relapse survival in ERα dependent breast cancer, and modulates ERα expression *in vitro*. *Int J Environ Res Public Health* **13**(1),ijerph13010036 (2015).

218. Wińska, P., Skierka, K., Łukowska-Chojnacka, E., Koronkiewicz, M., Cieśla, J., Bretner, M. Effect of simultaneous inhibition of protein kinase CK2 and thymidylate synthase in leukemia and breast cancer cells. *Anticancer Res* **38**(8),4617–4627 (2018).

219. Wu, D., Sui, C., Meng, F., Tian, X., Fu, L., Li, Y., et al. Stable knockdown of protein kinase CK2-alpha (CK2α) inhibits migration and invasion and induces inactivation of hedgehog signaling pathway in hepatocellular carcinoma Hep G2 cells. *Acta Histochem* **116**(8),1501–1508 (2014).

220. Wu, S-Y., Lee, A-Y., Lai, H-T., Zhang, H., Chiang, C-M. Phospho Switch Triggers Brd4 Chromatin Binding and Activator Recruitment for Gene-Specific Targeting. *Mol Cell* **49**(5),843–857 (2013).

221. Xie, Z.C., Tang, R.X., Gao, X., Xie, Q.N., Lin, J.Y., Chen, G., et al. A meta-analysis and bioinformatics exploration of the diagnostic value and molecular mechanism of miR-193a-5p in lung cancer. *Oncol Lett* **16**(4),4114–4128 (2018).

222. Xu, W., Chen, Q., Wang, Q., Sun, Y., Wang, S., Li, A., et al. JWA reverses cisplatin resistance via the CK2-XRCC1 pathway in human gastric cancer cells. *Cell Death Dis* **5**(12),1–11 (2014).

223. Yamane, K., Kinsella, T.J. CK2 inhibits apoptosis and changes its cellular localization following ionizing radiation. *Cancer Res* **65**(10),4362–4367 (2005).

224. Yang, B., Yao, J., Li, B., Shao, G., Cui, Y. Inhibition of protein kinase CK2 sensitizes non-small cell lung cancer cells to cisplatin via upregulation of PML. *Mol Cell Biochem*. **436**(1–2),87–97 (2017).

225. Yang, H., Minamishima, Y.A., Yan, Q., Schlisio, S., Ebert, B., Zhang, X., et al. pVHL acts as an Adapter to Promote the Inhibitory Phosphorylation of the NF-κB Agonist Card9 by CK2. *Mol Cell* **28**(1),15–27 (2007).

226. Yang, H.J., Youn, H., Seong, K.M., Jin, Y.W., Kim, J., Youn, B. Phosphorylation of ribosomal protein S3 and antiapoptotic TRAF2 protein mediates radioresistance in non-small cell lung cancer cells. *J Biol Chem* **288**(5),2965–2975 (2013).

227. Yang, K.M., Kim, K. Protein kinase CK2 modulation of pyruvate kinase M isoforms augments the Warburg effect in cancer cells. *J Cell Biochem* **119**(10),8501–8510 (2018).

228. Yao, K., Youn, H., Gao, X., Huang, B., Zhou, F., Li, B., et al. Casein kinase 2 inhibition attenuates androgen receptor function and cell proliferation in prostate cancer cells. *Prostate* **72**(13),1423–1430 (2012).

229. Yde, C.W., Frogne, T., Lykkesfeldt, A.E., Fichtner, I., Issinger, O.G., Stenvang. J. Induction of cell death in antiestrogen resistant human breast cancer cells by the protein kinase CK2 inhibitor DMAT. *Cancer Lett* **256**(2),229–237 (2007).

230. Yefi, R., Ponce, D.P., Niechi, I., Silva, E., Cabello, P., Rodriguez, D.A., et al. Protein kinase CK2 promotes cancer cell viability via up-regulation of cyclooxygenase-2 expression and enhanced prostaglandin E2 production. *J Cell Biochem* **112**(11),3167–3175 (2011).

231. Yoo, J.Y., Choi, H.K., Choi, K.C., Park, S.Y., Ota, I., Yook, J.I., et al. Nuclear hormone receptor corepressor promotes esophageal cancer cell invasion by transcriptional repression of interferon-γ-inducible protein 10 in a casein kinase 2-dependent manner. *Mol Biol Cell* **23**(15),2943–2954 (2012).

232. Yoo, J.Y., Lim, B.J., Choi, H.K., Hong, S.W., Jang, H.S., Kim, C, et al. CK2-NCoR signaling cascade promotes prostate tumorigenesis. *Oncotarget* **4**(7),972–983 (2013).

233. Yu, M., Yeh, J., Waes, C.V. Protein Kinase CK2 Mediates Inhibitor-Kappa B Kinase and Aberrant Nuclear Factor-κB Activation by Serum Factor(s) in Head and Neck Squamous Carcinoma Cells Ming. *Cancer Res* **66**(13),6722–6731 (2006).

234. Yu, W., Ding, X., Chen, F., Liu, M., Shen, S., Gu, X., et al. The phosphorylation of SEPT2 on Ser218 by casein kinase 2 is important to hepatoma carcinoma cell proliferation. *Mol Cell Biochem* **325**(1–2),61–67 (2009).

235. Zhang, H.X., Jiang, S.S., Zhang, X.F., Zhou, Z.Q., Pan, Q.Z., Chen, C.L., et al. Protein kinase CK2α catalytic subunit is overexpressed and serves as an unfavorable prognostic marker in primary hepatocellular carcinoma. *Oncotarget* **6**(33),34800–34817 (2015).

236. Zhang, S., Yang, Y.L., Wang, Y., You, B., Dai, Y., Chan, G., et al. CK2α, over-expressed in human malignant pleural mesothelioma, regulates the Hedgehog signaling pathway in mesothelioma cells. *J Exp Clin Cancer Res* **33**(1),1–12 (2014).

237. Zhang, X., Yang, X., Yang, C., Li, P., Yuan, W., Deng, X., et al. Targeting protein kinase CK2 suppresses bladder cancer cell survival via the glucose metabolic pathway. *Oncotarget* **7**(52),87361–87372 (2016).

238. Zhang, Y., Beck, C.A., Poletti, A., Edwards, D.P., Weigel, N.L. Identification of phosphorylation sites unique to the B form of human progesterone receptor. In vitro phosphorylation by casein kinase II. *J Biol Chem* **269**(49),31034–31040 (1994).

239. Zheng, Y., McFarland, B.C., Drygin, D., Yu, H., Bellis, S.L., Kim, H., et al. Targeting Protein Kinase CK2 Suppresses Pro-survival Signaling Pathways and Growth of Glioblastoma. *Clin Cancer Res* **19**(23),6484–6494 (2013).

240. Zheng, Y., Qin, H., Frank, S., Deng, L., Litchfield, D.W., Tefferi, A., et al. ACK2-dependent mechanism for activation of the JAK-STAT signaling pathway. *Blood* **118**(1),156–166 (2011).

241. Zhou, B., Ritt, D.A., Morrison, D.K., Der, C.J., Cox, A.D. Protein kinase CK2α maintains extracellular signal-regulated kinase (ERK) activity in a CK2α kinase-independent manner to promote resistance to inhibitors of RAF and MEK but not ERK in BRAF mutant melanoma. *J Biol Chem* **291**(34),17804–17815 (2016).

242. Zhou, F., Xu, J., Ding, G., Cao, L. Overexpression of CK2β and XIAP are associated with poor prognosis of patients with cholangiocarcinoma. *Pathol Oncol Res* **20**(1),73–79 (2014).

243. Zou, J., Luo, H., Zeng, Q., Dong, Z., Wu, D., Liu, L.. Protein kinase CK2α is overexpressed in colorectal cancer and modulates cell proliferation and invasion via regulating EMT-related genes. *J Transl Med* **9**(1),1–11 (2011).

244. Perea, S.E., Reyes, O., Baladron, I., Perera, Y., Farina, H., Gil, J., et al. CIGB-300, a novel proapoptotic peptide that impairs the CK2 phosphorylation and exhibits anticancer properties both in vitro and in vivo. *Mol Cell Biochem* **316**(1–2),163–167 (2008).

245. Weinstein, J.N., Collisson, E.A., Mills, G.B., Shaw, K.M., Ozenberger, B.A., Ellrott, K. et al. The Cancer Genome Atlas Pan-Cancer Analysis Project. *Nat Genetics* **45**(10),1113-1120 (2013).

246. Edwards, N.J., Oberti, M., Thangudu, R.R., Cai, S., McGarvey, P.B., Jacob, S., et al. The CPTAC Data Portal: A Resource for Cancer Proteomics Research. *J Proteome Res* **14**(6),2707-2713 (2015).

247. Chandrashekar, D.S., Bashel, B., Balasubramanya, S.A.H, Creighton, C.J., Ponce-Rodriguez, I., Chakravarthi, B.V.S.K., et al. UALCAN: A Portal for Facilitating Tumor Subgroup Gene Expression and Survival Analyses. *Neoplasia*. **19**(8),649–658 (2017).
